# MARS: A Multipurpose Software for Untargeted LC–MS-Based Metabolomics and Exposomics

**DOI:** 10.1021/acs.analchem.3c03620

**Published:** 2024-01-18

**Authors:** Laura Goracci, Paolo Tiberi, Stefano Di Bona, Stefano Bonciarelli, Giovanna Ilaria Passeri, Marta Piroddi, Simone Moretti, Claudia Volpi, Ismael Zamora, Gabriele Cruciani

**Affiliations:** † Department of Chemistry, Biology and Biotechnology, Universita degli Studi di Perugia, via Elce di Sotto 8, Perugia 06123, Italy; ‡ Molecular Discovery Ltd., Centennial Park, Borehamwood, Hertfordshire WD6 4PJ, U.K.; § Molecular Horizon, Via Montelino, 30, Bettona (PG) 06084, Italy; ∥ Department of Medicine and Surgery, P.le Gambuli 1, Perugia 06129, Italy; ⊥ Mass Analytica, Rambla de celler 113, Sant Cugat del Vallés 08173, Spain

## Abstract

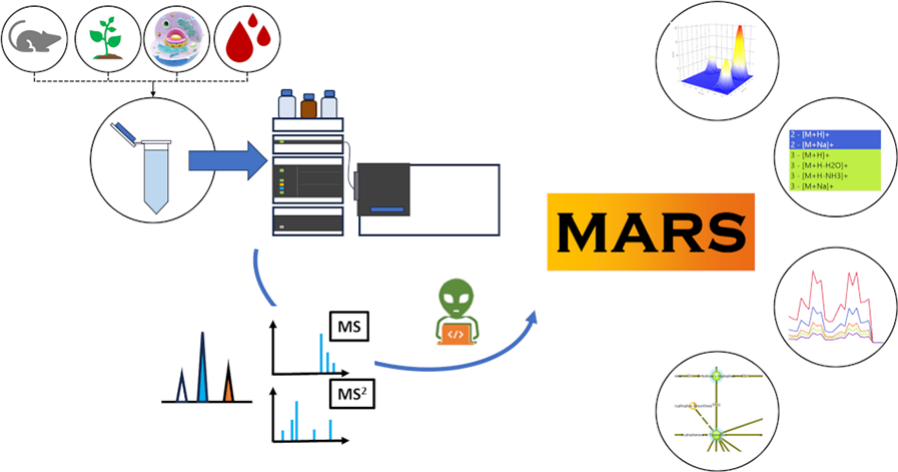

Untargeted metabolomics is a growing field, in which recent advances in high-resolution mass spectrometry coupled with liquid chromatography (LC-MS) have facilitated untargeted approaches as a result of improvements in sensitivity, mass accuracy, and resolving power. However, a very large amount of data are generated. Consequently, using computational tools is now mandatory for the in-depth analysis of untargeted metabolomics data. This article describes MetAbolomics ReSearch (MARS), an all-in-one vendor-agnostic graphical user interface-based software applying LC-MS analysis to untargeted metabolomics. All of the analytical steps are described (from instrument data conversion and processing to statistical analysis, annotation/identification, quantification, and preliminary biological interpretation), and tools developed to improve annotation accuracy (e.g., multiple adducts and in-source fragmentation detection, trends across samples, and the MS/MS validator) are highlighted. In addition, MARS allows in-house building of reference databases, to bypass the limits of freely available MS/MS spectra collections. Focusing on the flexibility of the software and its user-friendliness, which are two important features in multipurpose software, MARS could provide new perspectives in untargeted metabolomics data analysis.

Metabolomics has exploded in the last two decades and is now routinely used in clinics,^1^ pharmaceutical companies,^2,3^ the food industry,^4^ nutraceutics,^5^ environmental studies,^6^ forensic applications,^7^ among others. Indeed, a number of more focused branches have emerged under the general umbrella of metabolomics such as phytomics,^8^ exposomics,^9^ and lipidomics.^10^ Of these, lipidomics has recently been considered to be a stand-alone discipline due to the unique physical-chemical and structural features of lipids.^11^ Untargeted LC-MS-based analysis is widely used in metabolomics,^12^ and untargeted metabolomics can be defined as the study of the entire metabolite pool in a biological system.^13^ The main advantage of untargeted approaches is that they can also be applied at an early stage of research, and progress made in hardware and software development has recently boosted the use of untargeted workflows. However, untargeted approaches commonly generate a massive amount of data to be interpreted. Consequently, the number of in silico tools and software for untargeted metabolomics has increased in the past decade. At the time of writing several software programs and platforms are available,^14−17^ and guidelines for metabolomics software tools have also been proposed recently.^18,19^ In particular, Chang et al.^18^ commented that a graphical user interface (GUI) and the use of input/output files in standard format facilitate the widespread use of software that should cover at least basic workflows and provide a user-friendly installation system and documentation (i.e., manuals and tutorials). In addition, the authors emphasize that developing vendor-agnostic “plug-and-play” software simplifies access for less expert users. Among all of the usual steps in data analysis (data processing, statistical analysis, annotation/identification, functional analysis, and possibly quantification), metabolite annotation/identification in untargeted metabolomics represents one of the most critical steps and is a significant bottleneck.^20^ In lipidomics, a branch of metabolomics, analysis is facilitated by the consideration that each lipid subclass may contain thousands of lipid species sharing a common structure frame. Therefore, computational, rule-based tools for untargeted lipid annotation could be developed in lipidomics.^16,21−24^ In addition, other tools such as the Kendrick Mass Defect^25^ plot have proved to be very useful in lipidomics^26^ as they take advantage of the fact that several lipids in the same lipid subclass only differ by several repeating units (e.g., CH2). Chemical variability in untargeted metabolomics is greater than that in lipidomics, and compounds belonging to the same subclass according to the commonly used classification systems (e.g., Human Metabolome Database (HMDB)) do not always share a similar fragmentation pattern. As a result, rule-based approaches apply to a lesser extent, and in-house experimental libraries or publicly available databases (containing experimental and/or in silico generated data) are mainly used to identify features based on matching strategies.^27−29^ A large collection of data sets suitable for metabolomics is listed in the Metabolomics Workbench^30^ and databases for untargeted applications have already been reviewed elsewhere.^31^ Among the available databases, HMDB,^32^ MassBank of North America (MoNA),^33^ mzCloud,^34^ Metabolite and Tandem MS Database (METLIN),^35^ as well as LIPID MAPS Database^36^ and LipidBlast,^37^ which focus on lipids, are probably the most widely used. Unfortunately, freely accessible and downloadable databases can still suffer from some limitations, especially for untargeted identification.^38−40^ For example, the HMDB collection only contains MS/MS information on protonated and deprotonated species, and therefore different adducts are not identified by spectral matching approaches. Alternatively, in-house libraries can be built ad hoc for monitoring a defined data set of metabolites, and plates for high-throughput acquisition of LC-MS/MS data are already available on the market.^41^ In the latter case, software could help to automate the generation of a ready-to-use library. To broaden our studies from lipidomics to metabolomics, vendor-agnostic software for metabolomics endowed with a GUI called MetAbolomics ReSearch (MARS) was developed. This article describes its general software architecture and provides an in-depth description of the feature annotation module along with information about algorithms and tools from data processing to data analysis. Finally, a simple case study is provided to illustrate the software features.

## Workflow and Methods

**Figure 1:**
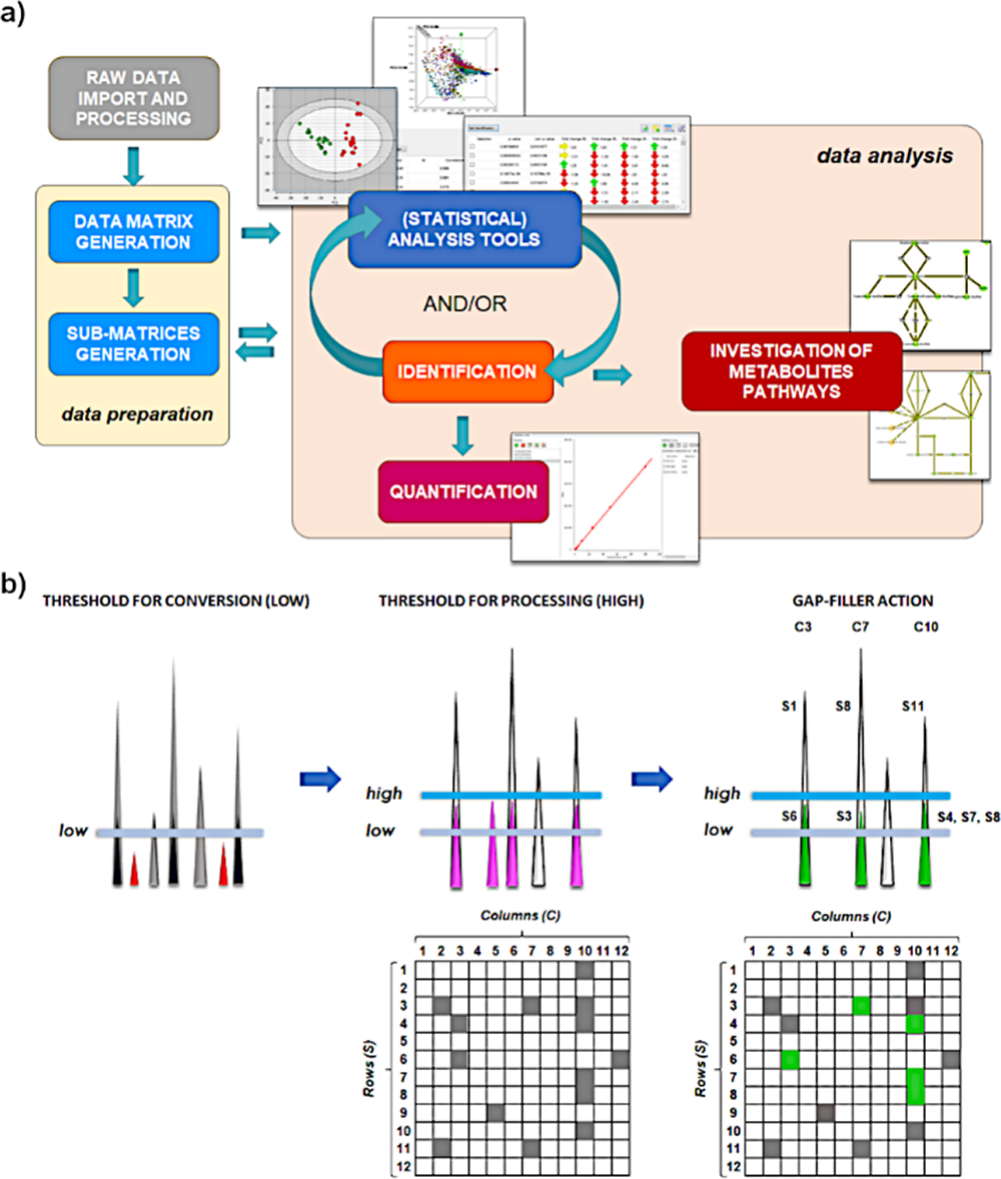
MARS architecture and the gap-filler algorithm. (a) General scheme of the MARS software. (b) Gap-filler algorithm. “Low” threshold for conversion (left): gray peaks are converted, while red peaks are removed in the import step according to a customizable threshold (low) and will not be available in the MARS session. “High” threshold for processing (center): white peaks (above this threshold) are processed and define the columns in the data matrix while the converted pink peaks are not processed being below the high threshold. Therefore, contrary to the red peaks, pink peaks are saved in the session but not used to define columns. Gap-filler action (right): green peaks, whose intensity is comprised between the two thresholds and that can refer to an empty cell in the data matrix, can be retrieved by the gap-filler.

MARS is a software developed in the C++ computer programming language with a GUI running on a Windows platform. The general scheme of the MARS software is shown in Figure 1a. Being vendor-agnostic software, instrument files are first converted and processed by MARS to generate a data matrix. It can be seen that data preparation and the data analysis moieties in Figure 1a are linked by a two-way arrow. Indeed, data analysis tools can also be applied to refine the data matrix (e.g., by selecting variables based on statistical analysis or identification results). The functions mentioned in Figure 1a are now described briefly. It was deliberately decided that MARS should share the general architecture of the latest version of Lipostar^21^ (Lipostar 2.1.2) so that the MARS sessions can also be opened in Lipostar 2 enabling lipids to be focused on without reprocessing instrument data. Nevertheless, MARS contains several specific tools for untargeted metabolomics, which makes MARS and Lipostar 2 two complementary software packages.

### Instrument Data Import and Processing

Instrument file formats acquired in full scan, data-dependent acquisition (DDA), and data-independent acquisition (DIA) approaches are supported for Agilent (.d), Waters (.raw), Thermo (.RAW), Sciex (.wiff), Bruker (.d), and Shimadzu (.lcd). More details are provided in Table S1. Default settings for each supported instrument are also provided for most of them, based on in-house testing of experimental instrument data. The default settings represent a starting point for further optimization by the user. Once converted and imported, instrument files are subject to additional data processing steps: (1) baseline and noise reduction, (2) peak extraction; (3) smoothing (Statistical Deconvolution Algorithm (SDA) or Savitzky-Golay^42^); (4) signal-to-noise ratio; (5) retention time (RT) correction; (6) alignment; (7) deisotoping; and (8) gap-filler (optional). It is noteworthy that MARS supports the processing of the ion mobility spectrometry (IMS) data, which was used in metabolomics because it enhances throughput and isomeric separation, and reduces chemical noise.^43,44^ For IMS data, a new peak detection algorithm is applied in MARS. Briefly, three-dimensional data (RT, drift time, and intensity) are extracted for each relevant feature (*m*/*z*), and the 3D peaks are generated. Each 3D peak is associated with the corresponding (pseudo-) MS/MS spectra when available. Compared to the processing used for non-ion mobility data, the algorithms for alignment, isotope clustering, gap filling, and identification in the new ion mobility processing consider the drift time dimension. When ion mobility data are processed, collisional cross section (CCS) or drift time (DT) values are also provided in the name of the detected features (the columns in the data matrix will be named using the *m*/*z*@RT(CCS or DT) format). In addition to IMS, an algorithm for Fourier-transform ion cyclotron resonance (FT-ICR) data is also available in MARS. Although MARS was designed for untargeted metabolomics, exclusive inclusion lists of *m*/*z*@RT or of *m*/*z*@RT(CCS or DT) can be used for feature detection in semi-targeted studies. Features are initially detected by considering all samples independently. Several additional algorithms can be applied after feature detection to align the samples, reduce the number of missing values, and recheck-area integration, which are discussed later.

#### RT Correction and Alignment

It is based on a list of standards or is carried out by promoting high-intensity features present in all samples as reference features in RT correction. By applying the second method, different intrabatch and interbatch RT tolerances can be applied to correct the batch effect using an algorithm based on the similarity of chromatograms.

#### Isotope Clustering

At this stage, isotopic features, as well as features related to different adducts of the same species, are yet to be clustered. After alignment, a column-wise search for isotopic patterns is carried out. The isotopic pattern relation was developed to consider theoretical spacing and is based on a series of chemical formulas compatible with the *m*/*z* value under investigation. As a result, a new matrix with isotopic patterns of grouped features is generated.

#### Gap Filler

Searching for low-abundance or low-response features represents a critical step in metabolomics. Indeed, lowering the processing threshold for signal intensity also increases the noise. On the other hand, strategies to reduce the noise may also remove low-abundance metabolites, increasing the number of missing values in the data matrix. When the gap filler is activated in MARS, the use of two independent thresholds for raw data file conversion and for processing allows the columns for data matrix generation to be selected from a higher threshold (the processing one), and then in a second step, the missing values to be reinspected, and the area for those peaks whose value lies between the two thresholds to be reimported (Figure 1b).

#### Peak Reintegration

Once the matrix has been generated, an optional algorithm allows the chromatographic peak “by column” to be reintegrated, increasing consistency.

### Data Matrix Generation and Refinement

Data processing leads to the generation of a data matrix in which samples are listed in rows and features detected in columns (Figure S1a). In MARS as in Lipostar,^21^ an extra-raw, named “Super Sample,” is calculated as a virtual pooled sample. Indeed, the Super Sample is composed of all features detected in at least one sample associated with an average area value for the chromatographic peak. In addition, the MS and MS/MS spectra are derived from all of the spectra experimentally available in samples by running an algorithm that discards noise signals. A 3D visualization can be activated when IMS data are analyzed (Figure S1b). MARS peak integration is fully automated, but the user can also manually reintegrate peaks in the data matrix if needed and the session is updated. In addition, total ion chromatogram (TIC) and extracted ion chromatogram (EIC) data can be visualized with no need to go back to the instrument (Supplementary Figure S2). Finally, in the case of missing MS/MS spectra, a tool to import newly acquired MS/MS spectra is also available for features of interest in the original instrument files used for matrix generation. Other operations are available to clean or refine the data matrix. They include (a) application of filters (e.g., blank subtraction, frequency filters); (b) normalization by metadata defined by the user (e.g., cell count, volume, weight) or by analysis related parameters (standards, total area, QC, etc.); (c) averaging over all replicates; (d) merging data matrices derived from the same samples acquired in positive and negative mode; and (e) adduct clustering (the latter operation after identification only).

Every time these operations are applied, new matrices are saved in a tree structure so that the user can compare results easily. Matrices generated represent the starting point of several operations, such as the use of statistical analysis tools, trend analysis, and identification. It should be noted that all generated matrices can be easily exported in .csv files (optionally including annotation results) so the user can analyze the data further using other tools.

### Analysis Tools

In addition to fold-change analysis and univariate statistical analysis (e.g., ANOVA), unsupervised and supervised multivariate statistical analysis tools are available in MARS. Concerning the supervised methods, in addition to the commonly used principal component analysis (PCA), the consensus PCA (CPCA)^45^ is also included. Indeed, although widely used, PCA can suffer when multiple influential factors are present. CPCA is a multi-block method designed to find the underlying relationships among several sets of possibly related data with an emphasis on revealing the “common trend” between these data. Although less frequently used, CPCA has been successfully used in metabolomics.^46^ PCA and PLS algorithms have also been enriched by the subspace discriminant index (SDI), a parameter designed for omics data that facilitate the inspection of a multicomponent model for data exploration by indicating the best component or pair of components to be inspected for effective discriminating any class of interest.^47^ The supervised methods PLS, PLS-DA, and O-PLS are available, together with linear discriminant analysis (LDA). The multivariate statistics module can also be accessed to process an externally generated data matrix by simply importing it as a .csv file, but in this case, several MARS functions that make the statistics plot linked to identification results or the pathways analysis will not work.

### Trend Analysis

Untargeted metabolomics represents a powerful tool in biomarker discovery because it can go beyond current knowledge and can also find the unexpected. Since metabolite annotation is currently a bottleneck in untargeted metabolomics,^20^ tools able to extract trends of interest for the features detected among the samples (including prior identification) enable a reduced set of metabolites to be focused on, reducing the time the annotation step takes. In this context, Geller et al.^2^ used trend analysis to refine multivariate statistical analysis outcomes (Figure S3a). To support trend analysis, MARS provides a tool to label the samples and uses the labeling to define trends. In this hypothesis-driven approach, the Pearson correlation coefficient is used to extract those features that match the trend according to a similarity threshold defined by the user (by default 0.8). Since this approach is based on labels applied by the user, the hypothesis-driven trend analysis finds applications in time-course experiments, monitoring of the disease course, and monitoring therapies, among others (Figure S3). The grouping-by-trend tool in MARS can also be used without predefining a desired trend by applying cluster analysis. In this case, the trend of each feature across the samples is generated, and commonly used clustering tools (K-means and Bisecting K-means)^48−50^ are applied. The output of these algorithms is a set of feature clusters. Features belonging to a particular cluster have similar trends, and the “quality” of each cluster is expressed as the percentage of the trends that are highly correlated to the average trend. Indeed, if this percentage is high, there is a high degree of coherence of trends in the cluster, and most of the trends are very similar (highly correlated) to many others. When the bisecting K-means algorithm is applied, the user can also evaluate the effect of splitting one cluster into 2 subclusters. Finally, MARS trend analysis based on the Pearson correlation coefficient can also be applied to find adducts or in-source fragments of the same metabolite. In this case, an RT tolerance parameter is used to limit the analysis to coeluting features.

### Identification of Metabolites

**Figure 2:**
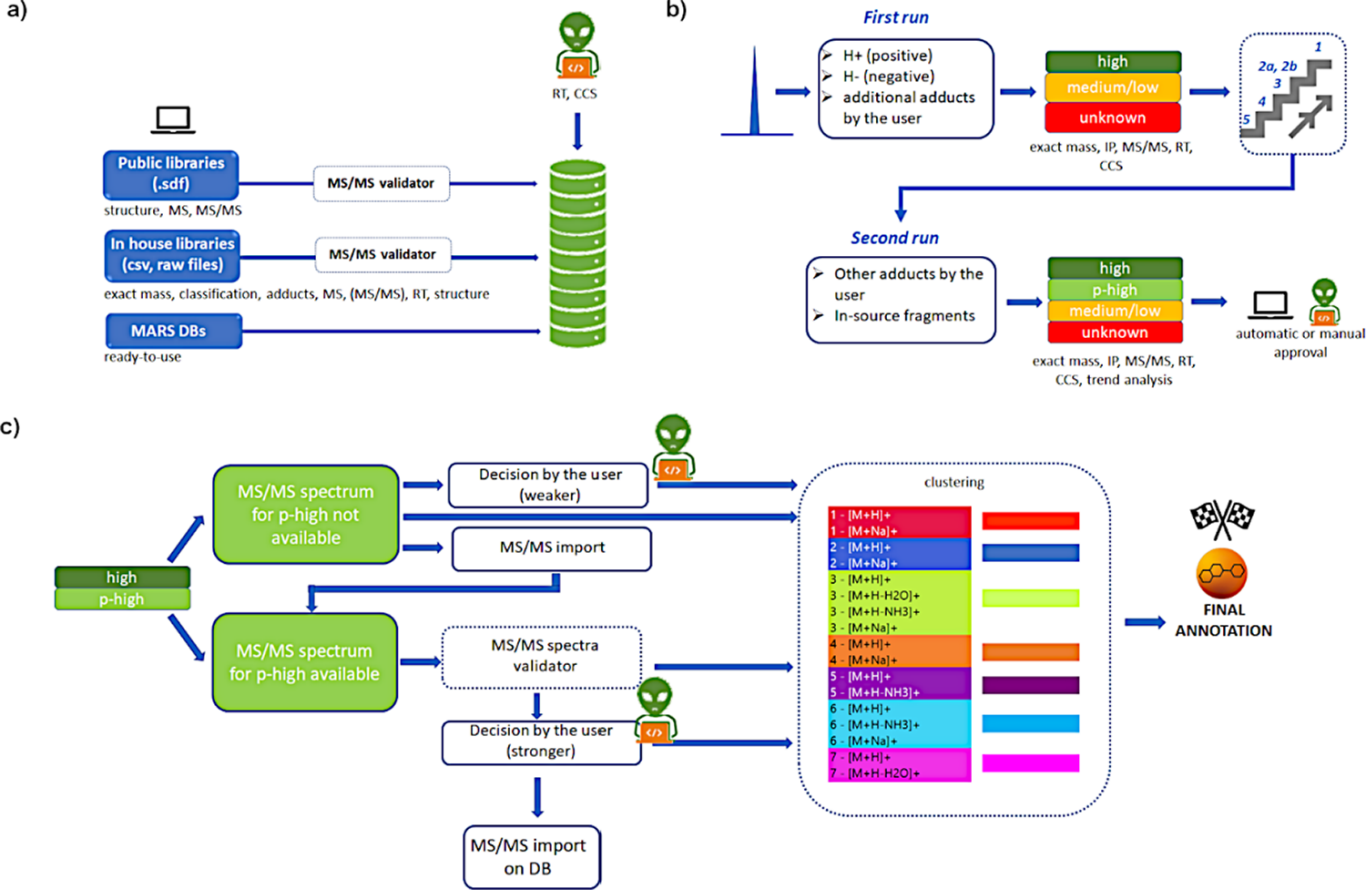
Scheme of the identification method in MARS is divided into three steps: (a) database generation through the DB Manager; (b) identification (in two runs); and (c) Refinement of the identification results, adduct clustering, and final annotation.

The scheme of the identification method in MARS shown in Figure 2 was devised. Three steps can be defined to describe this module: (a) database generation through the DB manager; (b) identification (in two runs); and (c) refinement of the identification results, adduct clustering, and final annotation. These steps are now described in detail.

#### Database Generation through the DB Manager

The identification module in MARS is based on the use of reference databases, to search for a mass match, and, when available, on MS/MS spectral matching with experimental or in silico predicted spectra and/or RT or CCS values. In this context, a separate executable of the MARS package, namely, the DB Manager, generates databases for identification purposes. Various data sources in the DB Manager can be used for data import and database generation (Figure 2a). Publicly available databases can be downloaded and used for data import in the DB Manager. This first version of MARS supports the import of the HMDB^32^ and MoNA. The recently developed Microbial Metabolites Database (MiMeDB)^51^ contains small molecule metabolites found in the human microbiome and is also compatible with MARS, being generated following the standard HMDB format. When the HMDB.sdf file is imported into the MARS DB Manager, experimental and/or predicted MS/MS spectra can be downloaded in as.xml format from the HMDB 5.0 Web site and linked to the corresponding metabolites in the DB Manager. A customizable threshold can be set by the user during the import of MS/MS in order to remove low-abundance signals close to the noise level. In addition, an algorithm named the MARS MS/MS validator can also be activated during the import of MS/MS spectra. This algorithm first fragments each structure in silico, and then the calculated fragments are used to recheck the imported spectra (see Supporting Information, paragraph 4-MS/MS validator section).^52^ As a result, potential noise or fragments not related to compounds of interest (not matched MS/MS signals) can be discarded, which consequently ensures a higher score during the identification process based on the spectral matching comparison. Obviously, the user can also manually assign additional fragments related to the compound if necessary, and these fragments can be used in future identification runs. Similarly, the NIST MoNa.sdf can be also uploaded. The user can add RT or CCS values, when available, at any time by using a .csv file or manually typing it in the GUI. There is an alternative way to build a MARS database that relies on importing in-house libraries starting from LC-MS or LC-MS/MS instrument data files (e.g., acquisition of standards, in-house experimental collections). A .csv file has to be prepared in order to provide the information needed for import, and the details are provided in the Supporting Information. Through this approach, the MARS in-house library is automatically generated with a minimum of user intervention. Nevertheless, several postprocessing operations are still available to the users who, using their experience, can manually add additional ionization adducts and associated fragment ions for a specific compound directly using the software GUI. Finally, ready-to-use, small, focused MARS databases have already been built, and they are available upon request. In this first version, two ready-to-use databases named MARS-phytoDB and MARS-naDB can be downloaded. The MARS-phytoDB contains flavonoids and phenolic, phenylacetic, and hydroxycinnamic acids from HMDB with rule-based predicted MS/MS fragmentation (26,068 compounds classified in 10 main classes and 75 subclasses). The MARS-naDB is a database of in silico generated nitrosamines with rule-based predicted MS/MS fragmentations downloadable for exposomics applications. This database comprises a total of 27,944 nitrosamines collected from various sources: safety assessments of pharmaceutical regulatory agencies, commercial suppliers, and in-silico-generated structures. Fragmentation rules were coded from the literature^53−55^ and in-house sources and the whole database was fragmented accordingly (more details on the two databases are provided in the Supporting Information).

Tutorials are available to guide less expert users with step-by-step instructions on how to generate databases. In addition, templates to help prepare the .csv files are available. It is worth noting that the MARS database can be modified and expanded by the user. Indeed, MARS allows the user to upload new information from the identification results to the database, and the MS/MS validator is also available to increase confidence. For example, although a given metabolite in the HMDB is associated with the MS/MS of the protonated form only, the user could link the MS/MS spectra for other adducts identified in a MARS session to the same entry. In addition, approved identification results of originally unknown metabolites can be easily added to the database for future identification runs.

#### Identification (in Two Runs)

The identification process in MARS occurs in two runs and relies on the information collected in the Super Sample (Figure 2b). Running the identification on the Super Sample has the advantage that the time identification takes only depends on the number of detected features, and not on the number of analyzed samples, making this approach very suitable for large data sets. As exemplified in Figure 2b, detected features are initially inspected by looking for protonated or deprotonated forms (depending on the acquisition mode) of metabolites included in the database. Additional adducts can be added by the user to this “first run list.” Once the first run was completed, all of the possible matches are scored. The overall score (OS) is the weighted average of four partial scores:
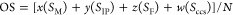
where *S*_M_, *S*_IP_, *S*_F_, and *S*_CCS_ are the mass score, isotopic pattern score, fragment score, and cross-collision section score, respectively, while *N* is the sum of the weights (*x* + *y* + *z* + *w*) (see the Supporting Information for details). By default, weights are set to 30, 10, 60, and 0 for *x*, *y*, *z*, and *w*, respectively, considering that ion mobility is currently less used in metabolomics. Default weights can be modified by the user to activate the CCS score as well as to increase or reduce the effect of other partial scores. A partial score for RT is not present in MARS; indeed, RT (when available) is used to filter out nonmatching compounds to reduce the false discovery rate. In addition to numerical scores, MARS uses visualization codes (colors and stars) to indicate the confidence level of each annotation. A summary is provided in Table 1.

**Table 1:**
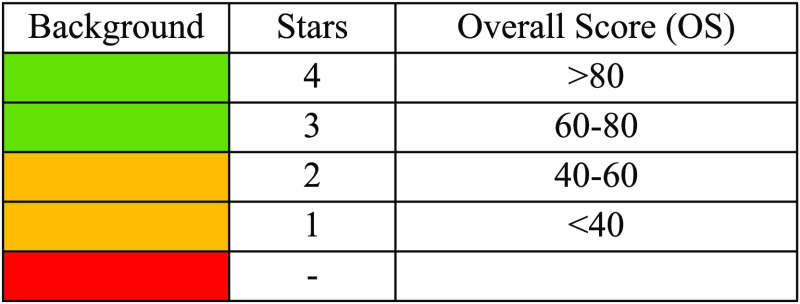
Summary of Confidence by Color-Based Annotation in MARS

Particularly, a green background is applied when the OS is greater than 60. By using default weights, the green color is usually displayed when a good MS/MS spectral matching is obtained. On the contrary, an orange background is applied to a lower overall score value (<60). Since the default *S*_F_ weight is much higher than the others, an orange background commonly appears according to various scenarios: (1) the MS/MS spectrum was acquired for a given feature, but the spectral matching approach provides very low *S*_F_ scores; and (2) the spectral matching approach is not applicable due to the lack of the MS/MS spectra in the DB and/or the lack of an acquired MS/MS spectrum for a given feature. Finally, a red background is used when there are no matches by *m*/*z* values within the applied tolerance (unknown features). Within the color-based classification, stars provide immediate information about the overall score range. When more than one solution is possible according to the identification criteria used (e.g., tolerance in exact mass and RT range), solutions are ranked by the global score. When isobars are possible, they are displayed with either the same score or a different score based on the absence or presence of discriminant fragments, respectively. In addition, MARS is endowed with an optional level-based classification similar to the Shymanski classification,^56^ which is largely used as a standard index of annotation confidence, with minor adaptation as shown in Table S2. Once the first run has been completed, a second run is automatically carried out to inspect coeluted peaks for each feature identified by the first run in order to detect other possible adducts and in-source fragments (Figure 2b). In the identification method, the user is asked to select which adducts and in-source fragmentations to inspect in the second run. During the second run, the check is based not only on the compatible *m*/*z* value and RT but also on the isotopic pattern and the trend across the samples (adducts and in-source fragments must have a similar trend across the samples). Due to the second run, features previously labeled with lower scores (orange labeling) or still unknown (red labeling), but now identified, are promoted to a light-green color (promoted-high or p-high confidence). All of the information obtained is available to the user, who may then decide to use either the automatic approval feature or manual revision.

#### Refinement of the Identification Results, Adduct Clustering, and Final Annotation

Additional tools are available to further inspect the high-p annotations (Figure 2c). Indeed, the p-high-labeled features may or may not have experimental MS/MS spectra. When MS/MS spectra are not available for these promoted species, the user has three options: (1) a decision about their approval can be made by the user based on previous knowledge; (2) the solution/s is/are automatically approved, with no further detection (more risky); and (3) samples are reanalyzed with an inclusion list to force the MS/MS acquisition for the features of interest. Therefore, the new MS/MS spectra are imported into the MARS session to use the MS/MS validator for confirmation. When the MS/MS spectra are available, the MS/MS validator can be directly applied for confirmation, or manual interpretation can be performed. What is more, the MS/MS validator tool will be discussed further in the case study in the second part of this manuscript. The MS/MS spectrum of a metabolite annotation approved by the user can also be added to the MARS database in order to improve identification in future runs (Figure 2c). Once all features have been automatically and/or manually approved, the adduct/in-source fragment clustering algorithm can be launched for final annotation. In particular, different adducts of the same molecule are clustered together if their RTs differ within a narrow user-defined range and if they show a similar trend in the acquired samples (optional request). In-source fragmentations can arise from the loss of small neutral molecules (e.g., H_2_O and NH_3_) but can also derive from the loss of larger chemical moieties (e.g., ribose, ribose mono-, di- triphosphate, deoxyribose, etc.). If the search of the first ones can be simply set in the identification method, it would be challenging for the user to include all potential in-source due to the great chemical variability of the metabolome. Therefore, MARS provides an additional tool that inspects all chemical features that fall within the desired RT tolerance. When two features coelute, if the one with the higher *m*/*z* value is endowed with MS/MS spectra, this tool will search for the parent-ion signal of the feature with a lower *m*/*z* value within the MS/MS spectrum of the first compound and is considered to be an ISF feature during adduct and ISF clustering.

### Stable Isotope Labeling Studies

The MARS identification module also provides tools for stable isotope labeling studies. First, it is well-established that molecular formula assignment can be facilitated in cell-culture studies where cells can be grown on a uniformly unlabeled (12C-) or uniformly labeled (13C-) carbon source.^57^ Indeed, the number of carbon atoms for a metabolite at a given RT will be defined by the mass difference between labeled and unlabeled species that will coelute, and this can drastically reduce the search space for possible molecular formulas.^57^ Therefore, a database of unlabeled species in the MARS DB Manager can be automatically converted to a fully 13C-labeled database in a single click. In addition to the new exact mass, the MS/MS fragmentation can also be recalculated. The only requirement is that during the database generation in the DB Manager, the MS/MS validator tool is activated to allow the collection of the elemental composition associated with each MS/MS fragment. When a database of uniformly labeled or unlabeled species is used for identification, MARS uses the match between the two coeluting isotopologues to reduce the molecular formulas associated with a given feature. In addition, MARS DB Manager allows the addition of other labeled species through the manual editing of a structure present in the database by selecting which atoms should be labeled and saving this as a new structure.

### Pathway Analysis

MARS in this first version is endowed with a collection of 20 metabolomics pathways (see the Supporting Information for the complete list) to allow the projection of identification results for functional analysis when a database with HMDB ID codes for entries is used. To follow a well-established classification, the available pathways are named based on the KEGG database. Each pathway map is a molecular interaction/reaction diagram consisting of nodes and edges, where nodes represent molecules and enzymes (different colors for human or non-human enzymes), while edges depict molecular interaction, reaction, and relation networks. The information needed to build the MARS pathways was obtained by integrating data from different online sources (KEGG metabolic network^58^ and PathBank pathways linked to HMDB)^59^ and sources in the literature. The list of the pathways and examples of added new literature data are provided in the Supporting Information.

### Quantification

Absolute quantification of metabolite concentrations is difficult to achieve in untargeted metabolomics. Therefore, the literature mostly reports relative changes and semiquantitative data. Indeed, untargeted metabolomics workflows often focus on detecting changes in the metabolome upon natural or induced perturbations, and so relative quantification is usually performed.^60^ Relative and absolute quantification using external and/or internal standards is supported in MARS. Calibration curves for absolute quantification can be automatically generated in MARS from raw acquisition files and can be edited, if necessary (e.g., point exclusion, integration check), and assigned to the species of interest that need to be quantified.

### Tool for Exposomics: Searching for Metabolites of Xenobiotics

Exposome is a term used to describe the totality of environmental exposures and lifestyle factors while exposomics is the science revealing the sheer amount of chemicals humans are exposed to, and identifying disrupted metabolic pathways.^61^ In pharmaceutical research, drugs are xenobiotics to which cells, animals, and humans are exposed in order to evaluate the biological response in common metabolomics pathways. Therefore, this kind of study is a particular case of exposomics in which the xenobiotic is deliberately selected to alter a metabolic state. However, xenobiotics in a living cell or organism undergo metabolic transformations, and therefore, searches for metabolism need to be included in the analysis. In MARS, it is possible to perform a preliminary analysis to search for the presence of potential metabolites of exogenous compounds in the samples under investigation. Indeed, a tool is available to specify one or more compounds of interest (imported in .sdf format) and to select a series of enzymatic and non-enzymatic reactions. Based on this information, a list of potential metabolites will be generated and the corresponding *m*/*z* values for a selection of adducts will be searched in the samples and labeled as potential metabolites of the compound(s) of interest (Figure S4). Far from being a comprehensive software for metabolite identification like Mass-MetaSite,^52^ this simple tool can provide useful insights to run more extensive analyses in a later stage.

## Case Study

**Figure 3:**
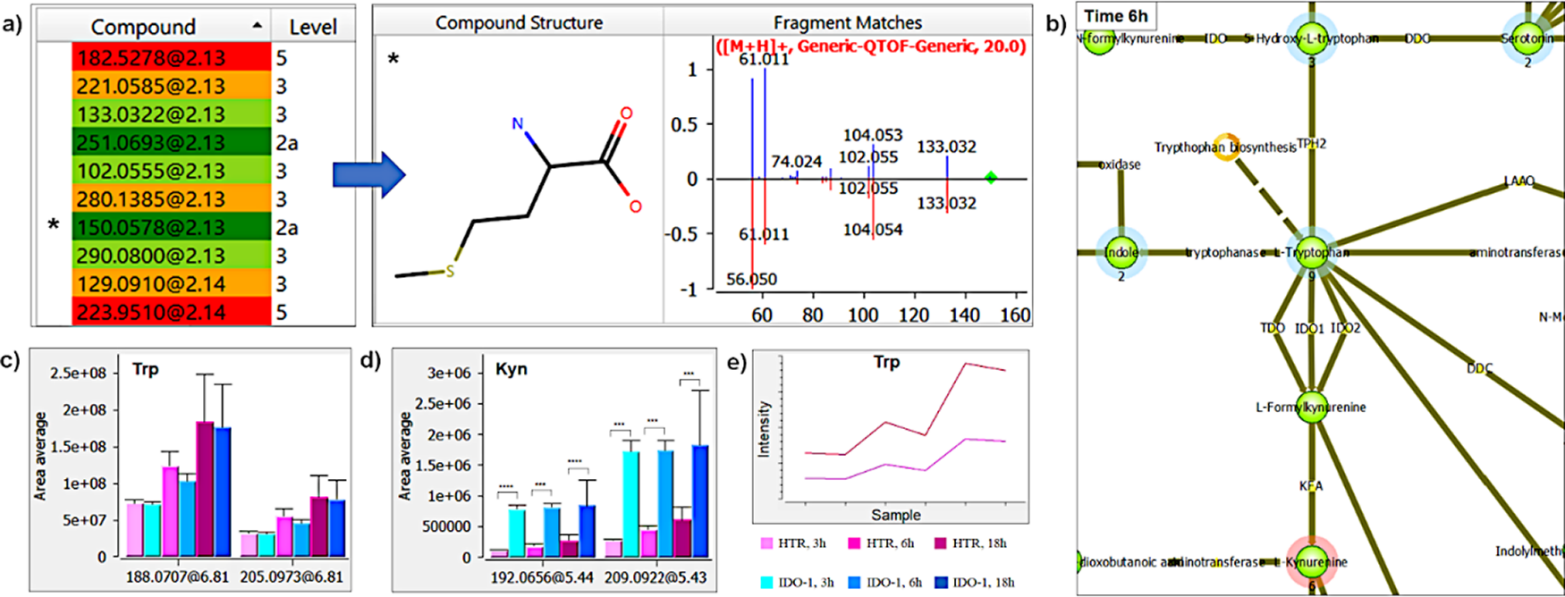
Selection of tools for data analysis. (a) Table of the identification results with color- and level-based confidence for each feature. Additional information on the identification (compound structure and fragment matches) is shown for the feature 150.0578@2.13 (*). (b) Pathway analysis for Trp and its metabolites at 6 h. Decreased (blue) and increased (red) species were defined by comparing P1.IDO1 samples to P1.HTR samples; (c) area plot for Trp detected as [M + H]^+^ at *m*/*z* 205.0973 (RT = 6.81 min) and as [M – NH_3_ + H]^+^ at *m*/*z* 188.0707 (RT = 6.81 min); and (d) area plot for Kyn detected as [M + H]^+^ at *m*/*z* 209.0922 (RT = 5.43 min) and as [M – NH_3_ + H]^+^ at *m*/*z* 192.0656 (RT = 5.44 min). Statistical significance: * (*P* ≤ 0.05), ** (*P* ≤ 0.01), *** (*P* ≤ 0.001), ****(*P* ≤ 0.0001); (e) trend analysis for Trp detected as [M + H]^+^ (purple), and as [M – NH_3_ + H]^+^ (dark red).

The aim of this case study is to illustrate several MARS features in practice, and it is not intended as an optimized experiment for biological interpretations. Indoleamine 2,3 dioxygenase 1 (IDO1) is a heme-containing cytosolic enzyme that acts on multiple substrates, including d-tryptophan, L-tryptophan (Trp), 5-hydroxy-tryptophan, tryptamine, and serotonin.^62^ Nevertheless, its most studied function is the conversion of the essential amino acid Trp to Kynurenine (Kyn), which represents the first, rate-limiting step in the so-called “Kynurenine pathway.”^63^ The activation of the enzymatic function of IDO1 along this pathway leads to Trp depletion and the production of a series of bioactive metabolites, collectively known as “kynurenines.” Both Trp starvation and kynurenine production represent fundamental immunoregulatory mechanisms and both are involved in the maintenance of immune homeostasis but are also crucial for neuronal function and intestinal homeostasis.^64,65^ P1.HTR cells represent a highly transfectable clonal variant of the mouse mastocytoma P815,^66^ and these cells have no basal expression of IDO1. In this case study, P1.HTR cells overexpressing IDO1 (P1.IDO1) and mock control cells (P1.HTR) were washed and then incubated for 3, 6, and 18 h in a medium containing Trp (16 mg/L). Experiments were performed in triplicate. Thus, a metabolomics analysis was carried out on the metabolite extracts of P1.IDO1 and P1.HTR at various incubation times. Thus, this small data set is here used to discuss potential solutions in MARS for more accurate annotation of metabolites, as well as a tool for preliminary biological interpretation. With the settings used (see Supporting Information) MARS initially detected 14,816 features (*m*/*z*@RT values). After blank subtraction and filtering out the features that were present in less than 50% of the samples, the matrix contained 8,073 features, whose 970 had MS/MS data. In this study, the HMDB database was used for spectral matching as it is easily accessible to a non-expert user. However, it is known that the MS/MS spectra collection in the HMDB is rather limited. At the end of the automatized metabolite annotation, 1,809 features were matched at least by exact mass (5 ppm tolerance), with various confidence levels (1–3). Concerning the stars-based confidence levels, features were classified as follows: 1 star: 1,586, 2 stars: 33, 3 stars: 44, and 4 stars: 146. As previously mentioned, MARS can facilitate the detection of ISF products. For instance, Tyrosine was identified as green/2a at RT 2.98, with several coeluting features (Figure S5a). The use of the adduct and ISF detection tool allowed the annotation of 4 ISF products of Tyrosine. In addition, the trend analysis confirmed the same trend across the samples (Figure S5b). Another important tool for curated annotation is the MS/MS validator. For instance, for the feature 238.1074@10.41, MS/MS data were acquired, but the reference database returned only eight potential matches based on exact *m*/*z*; thus, although the *S*_M_ score was 100 for all the potential annotations (Δppm = 0), the OS score was only 30 (orange/level 3/1 star) because ambiguous. By applying the MS/MS validator, the number of potential solutions was reduced to two endogenous metabolites, 1-carboxyethylphenylalanine and *N*-lactoylphenylalanine (Figure S6). Indeed, MARS contains the same in silico fragmentation engine common to Mass-MetaSite, an established software solution used in numerous pharma companies for drug-metabolite identification based on LC-MS data. Validation of the fragmentation tool has been already reported elsewhere.^52^ We also mentioned that MARS allows projecting the identification results (Figure 3a) onto the Trp pathway. When this operation is performed, the resulting nodes in the MARS maps that are populated are surrounded by a gray shadow; if two labels are selected to define a comparison criterion, the shadows will be colored in red or blue, meaning increased or decreased species, respectively. Figure 3b shows part of the Trp pathway in MARS after projection of the detected metabolites for 6h incubation samples. As expected, the increase in Kyn concentration observed (red shadow surrounding the corresponding node) is accompanied by a decrease in Trp concentration (blue shadow), which serves as a substrate for the production of Kyn itself along the Kynurenine pathway as well as in the species that would be produced by the conversion of Trp along different metabolic pathways, such as 5-hydroxy-tryptophan (produced from Trp along the Serotonin pathway) and indole. Overall, looking at the biochemical interpretation of the data, they confirm that the overexpression of the enzyme IDO1 in P1.IDO1 cells causes a shift in Trp metabolism toward the production of Kyn, the major product of its enzymatic activity. Another option is to plot the area of the peak for each potential feature of interest (Figure 3c, d), thereby evaluating the trend (Figure 3e). This visualization option shows that the catalytic activity of IDO1 is already detectable after 3 h of incubation, especially when attention is devoted to the production of Kyn- (Figure 3d). Since in this case study, we analyzed the content of the intracellular metabolites, the overall concentration of the internalized Trp increases over time both in P1.HTR and in P1.IDO1 cells (Figure 3c, e). Additional information on MARS performances including the feature overlap with other freely available software tools, and information on processing time when MARS is applied on larger data sets and or for the import of the whole “all spectra” MoNA database are available in the the Supporting Information (paragraph 6).

## Conclusions

This article describes MARS, vendor-agnostic software provided with a GUI that was designed for untargeted metabolomics by LC-MS analysis. MARS handles all of the steps of metabolomics analysis, including instrument data conversion and processing, statistical analysis, annotation/identification, quantification, and preliminary biological interpretation. Ion mobility data are also supported with the potential use of CCS values for annotation and a tool for 3D visualization and peak reintegration. Metabolite annotation in untargeted metabolomics is usually performed by querying databases, which have various natures and may or may not contain MS/MS data. Several ways to generate a reference database for annotation are possible in MARS such as the use of publicly available repositories and in-house-generated libraries. The latter option is probably the most expensive but the most robust strategy to overcome the lack of MS/MS information in publicly available databases. In addition, the MARS reference database can be trained by the user while working. Other innovative features include the 2-runs workflow in identification to detect adducts and in-source fragments not present in the database, the MS/MS validator to validate MS/MS fragmentation in silico, and (optionally) clean experimentally noisy MS/MS spectra from public libraries as well as the conversion of unlabeled databases to fully isotope labeled databases. In addition, two premade databases fragmented by applying fragmentation rules derived from the literature and experimental evidence are available on request for a selection of natural products and for nitrosamines. Biological interpretation is also facilitated since the annotated features can be projected on 20 metabolic maps available in MARS. Finally, a tool is available to detect potential metabolites of exogenous compounds based on a series of enzymatic and nonenzymatic reactions. Designed for exposomics, it can be applied to any compound present in the samples, including those used for cell treatment. Although this tool is not intended as comprehensive software for metabolite structure elucidation, it can provide useful insights to drive further investigation. Applying the recommendations of Chang et al.^18^ makes the GUI easy to use for nonexpert users, and advanced settings are also available for more expert users. MARS is also provided with a manual and 12 tutorials. Furthermore, data matrices generated in MARS are exportable as .csv files as well as annotation results. It is noteworthy that MARS sessions are fully compatible with the Lipostar software,^21^ to combine metabolomics and lipidomics analysis with no need to reprocess data. In the opinion of the authors, the MARS architecture and the versatility of its use may contribute to broadening the application of untargeted metabolomics workflows.

## Supporting Information Available

Further description for MARS features; rule-based databases; MARS processing performances; experimental details for the case study (cell-based assay, extraction of metabolites, LC–MS method, and Data Analysis) (PDF)
