# Supplementary material for: MARS: A Multipurpose Software for Untargeted LC–MS-Based Metabolomics and Exposomics
Source: Anal Chem. 2024 Jan 18;96(4):1468–77. doi: 10.1021/acs.analchem.3c03620 (PMC10831794; doi:10.1021/acs.analchem.3c03620)
Supplement: Supplementary file 1 — ac3c03620_si_001.pdf [file ac3c03620_si_001.pdf]

Supporting Information

MARS: A Multipurpose Software for Untargeted LC-MS-Based Metabolomics and Exposomics

Laura Goracci,[1‡] Paolo Tiberi, [‡2] Stefano Di Bona,[3] Stefano Bonciarelli,[2] Giovanna Ilaria Passeri,[2] Marta Piroddi,[3] Simone Moretti,[3] Claudia Volpi,[4] Ismael Zamora,[5] Gabriele Cruciani[1]*

[1]Department of Chemistry, Biology and Biotechnology, via Elce di Sotto 8, 06123, Perugia, Italy.

[2]Molecular Discovery Ltd., Centennial Park, Borehamwood, Hertfordshire, WD6 4PJ United Kingdom

[3]Molecular Horizon, Via Montelino, 30, 06084 Bettona (PG), Italy,

[4]Department of Medicine and Surgery, P.le Gambuli 1, 06129 Perugia, Italy

[5]Mass Analytica, Rambla de celler 113, 08173, Sant Cugat del Vallés, Spain.

laura.goracci@unipg.it

Table of contents

1. Supplementary Figures S3
2. Supplementary Tables S8
3. Downloadable Rule-based databases S9
DB for phytomics application (MARS-phytoDB) S9
DB for nitrosamine detection (MARS-naDB) S9
4. Further details on MARS algorithms and tools S10
Template for the generation of a reference DB from in-house data S10
Pathway analysis S10
Identification Score Equations S11
5. Supplementary material CASE STUDY S12
Cell based assay S12
Extraction of metabolites S13
LC-MS method S13
Data Analysis using MARS S14
6. Further information about MARS performances S14
Feature detection overlap among different software S14
Import of the MoNA (all) database in the MARS DB Manager S15
Time required to detect features in one sample S16
Processing data on a large study S16

1. Supplementary Figures

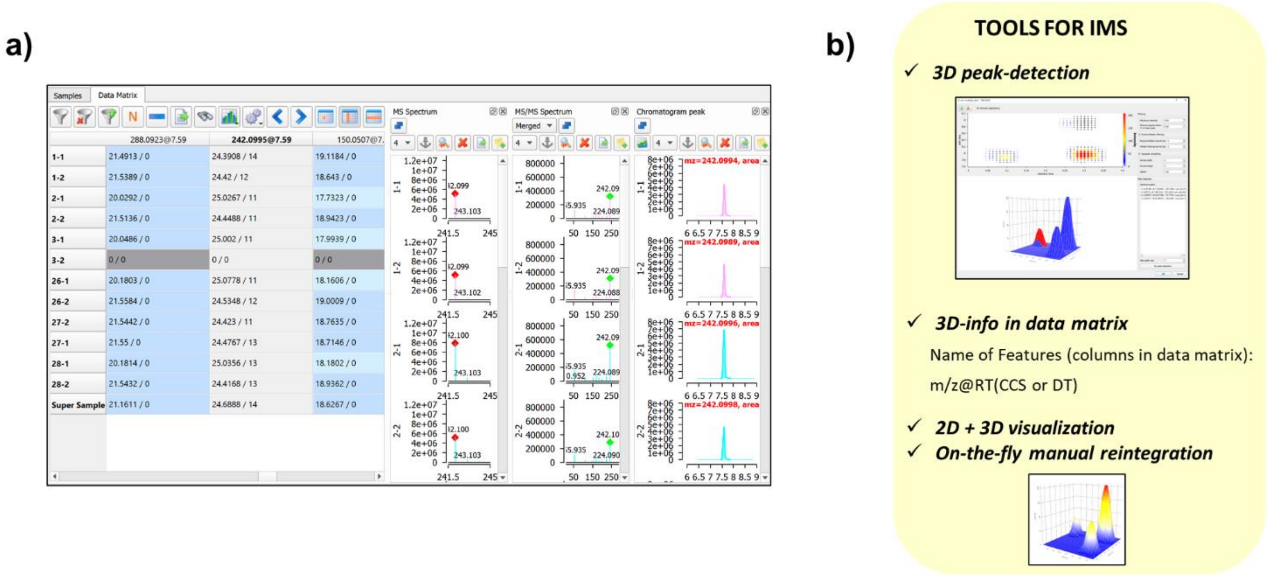

Figure S1. Overview of the MARS Graphical User Interface for inspecting the data matrix. a) The Data Matrix tab showing the data matrix generated; b) Additional tools available for detection and visualization of ion mobility data.

a)

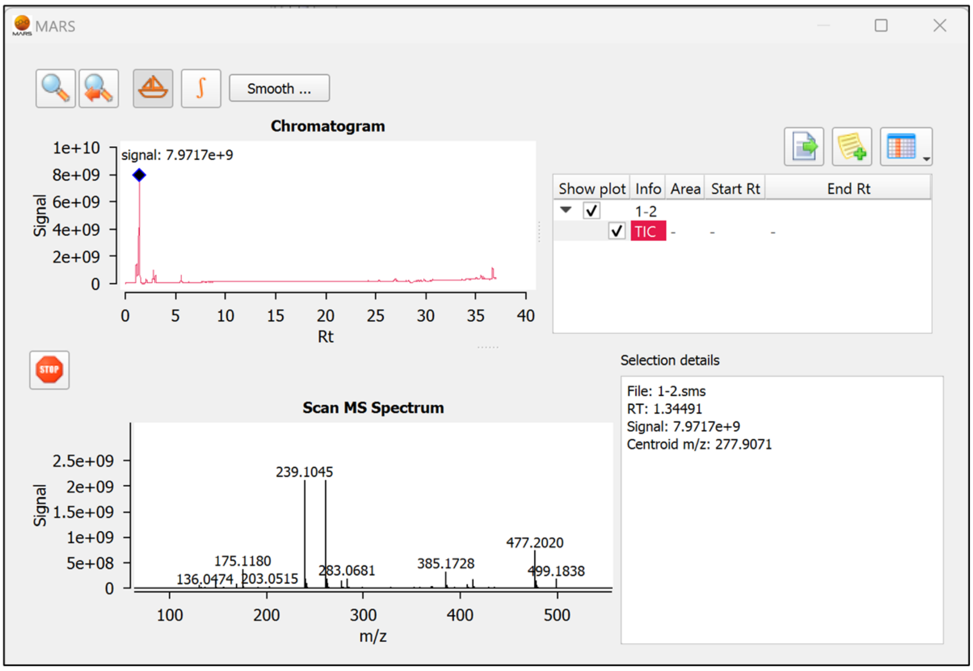

b)

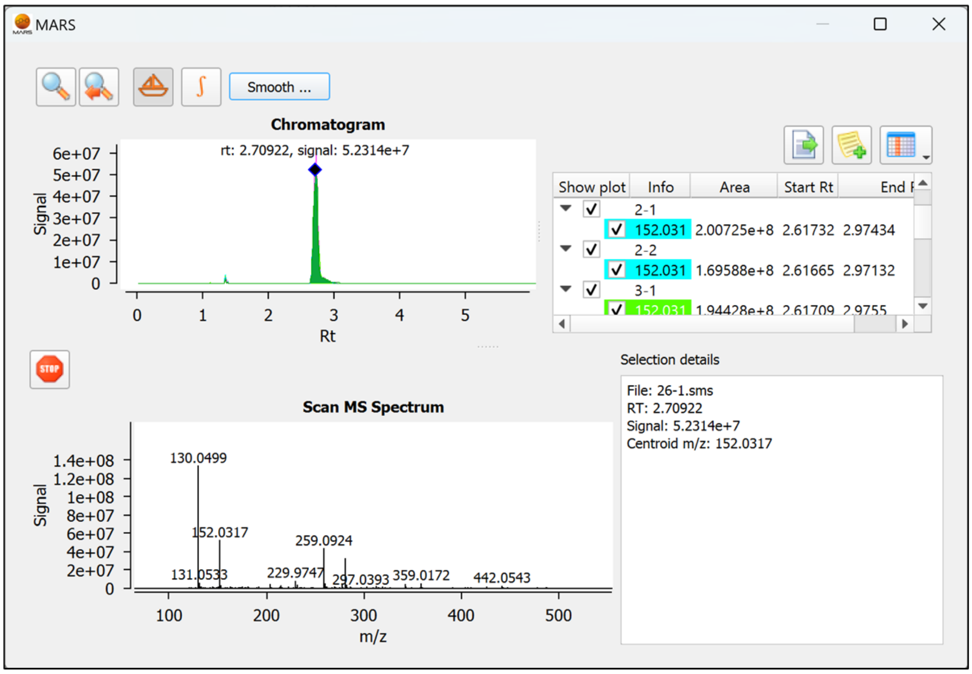

Figure S2. Tool for a) TIC and b) EIC inspection and for manual peak integration.

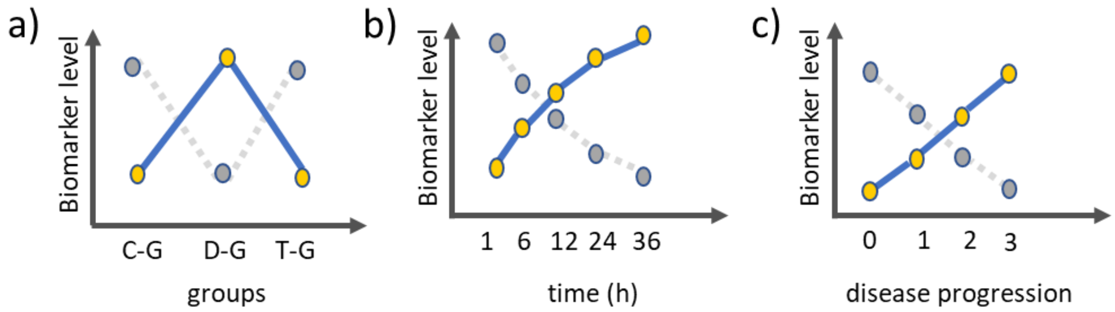

Figure S3. Examples of trends across samples for biomarker discovery. Gray-dotted trends represent the inversely correlated behaviors, which are still good trends. a) Example of expected trend in the example mentioned in Geller et al.[1] Three groups of samples were compared in their study on monitoring the effect of a drug treatment: healthy sample control group (CG), diseased group (DG), and disease-treated group (TG). Therefore, any feature endowed with an elevated or reduced mean peak height in DG compared to the CG and subsequently corrected in the TG could be considered to be a potential biomarker; b) example of a time-course trend; c) example of a disease progression trend based on different levels in a disease scale.

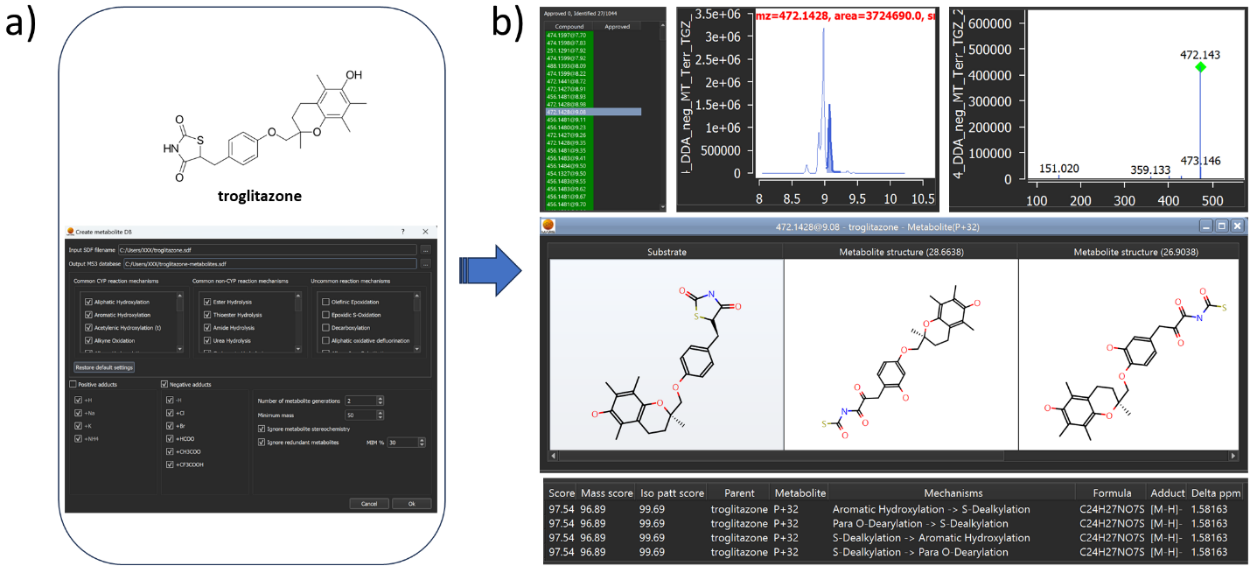

Figure S4. Scheme of the tool to search for metabolites of xenobiotics applied to troglitazone, here shown by visualizing MARS in his dark visualization mode. a) A library of in silico predicted metabolites of troglitazone is generated by combined structural information of the substrate (.sdf format) and a library of metabolic reactions. b) Output generated by MARS, which includes a list of potential metabolites, chromatographic peak, MS/MS spectra, potential structures compatible with the given peak and the list of reaction(s) that can play a role in the metabolite's formation. Isotopic pattern can be also displayed. Differently from Mass-MetaSite, the interpretation of the MS/MS fragmentation for complete MetID is not available in MARS.

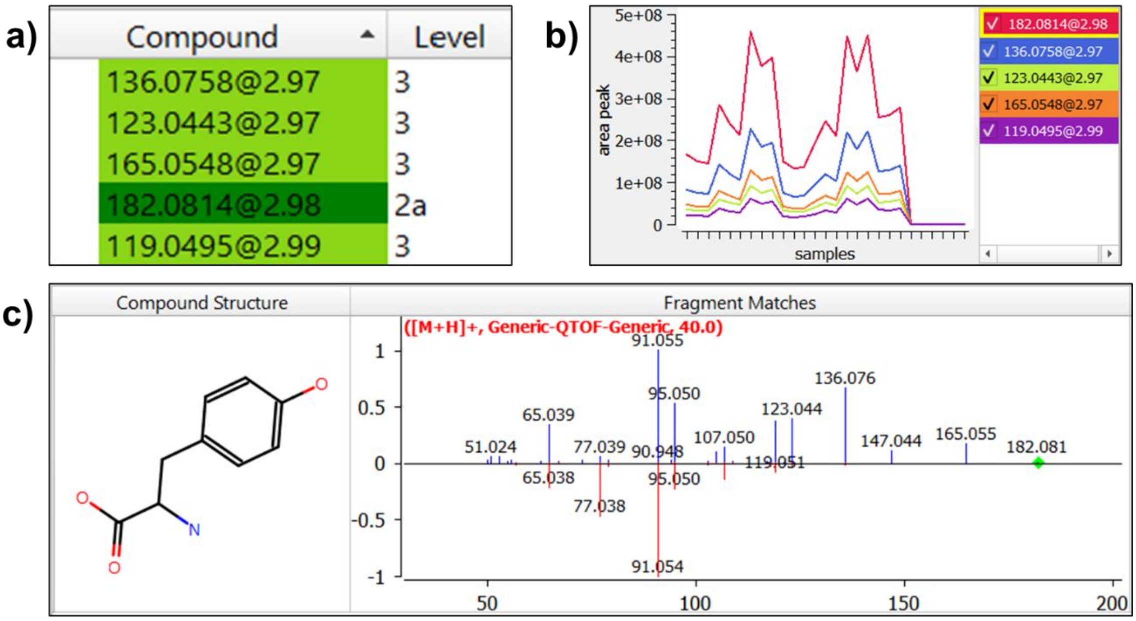

Figure S5. Automatized detection of Tyrosine in-source fragmentation products. a) Tyrosine (182.0814@2.98) is identified as [M+H]+ with confidence level 2a and four potential in-source fragmentation products of Tyrosine are detected; b) the trend analysis is used here to show that the features in (a) are highly correlated, showing the same trend among the samples; c) the inspection of the experimental MS/MS spectra of Tyrosine clearly shows that the m/z values of coeluting species in (a) correspond to those of the high-intensity fragments (m/z 136.0758, 123.0443, 165.0548, and 119.0495). This information check is part of the ISF clustering algorithm.

a)

b)

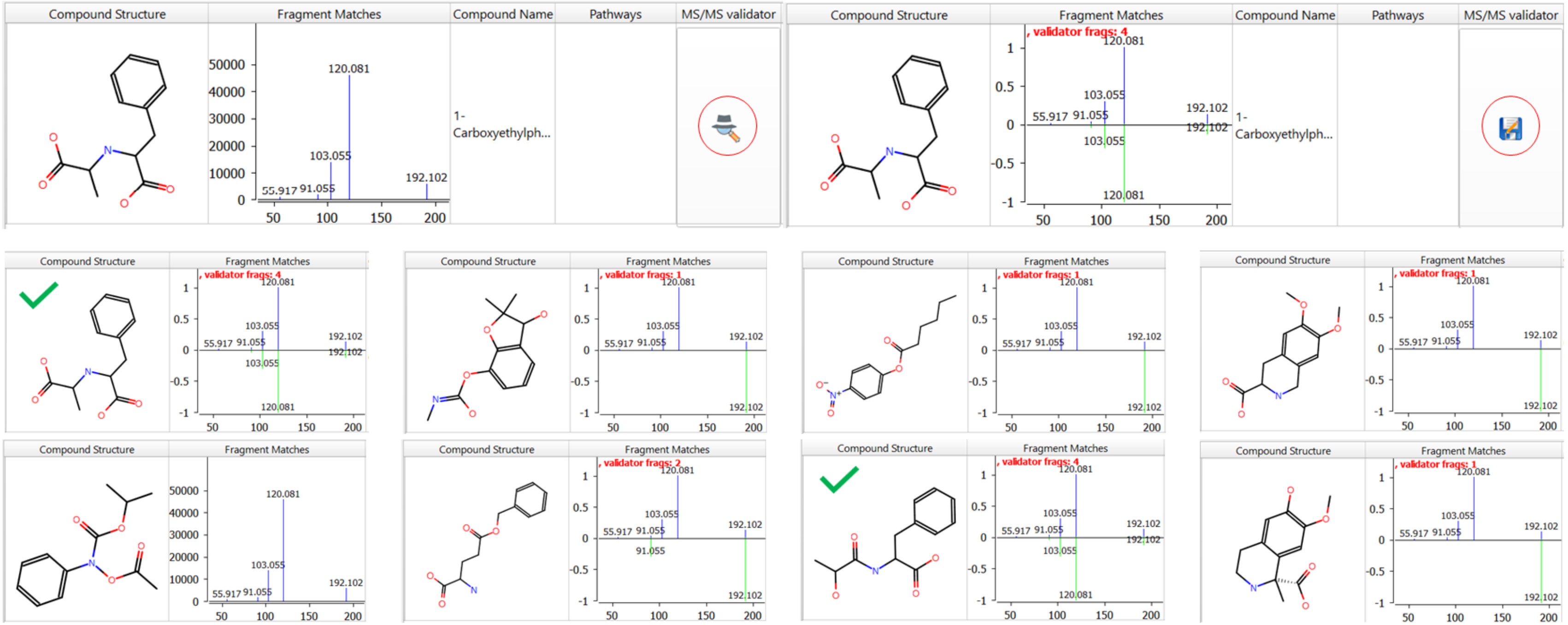

HMDB ID	Stars	OS	S_M	S_{IP}	S_F	SC_{CCS}	CCS	Num Frag Matches	Name	Formula	Compound Class	Mass	Mass delta ppm	Adduct
HMDB0240628	1	30	100	0	0	0	0	0	1-Carboxyethylphenylalanine	C12H15NO4	Amino acids, peptides, and analogues	237.1001	0	[M+H]+
HMDB0032775	1	30	100	0	0	0	0	0	3-Hydroxy-carbofuran	C12H15NO4	Coumarans	237.1001	0	[M+H]+
HMDB0246542	1	30	100	0	0	0	0	0	4-Nitrophenyl hexanoate	C12H15NO4	Phenol esters	237.1001	0	[M+H]+
HMDB0247110	1	30	100	0	0	0	0	0	6,7-Dimethoxy-1,2,3,4-tetrahydroisoquinoline-3-carboxylic acid	C12H15NO4	Tetrahydroisoquinolines	237.1001	0	[M+H]+
HMDB0247993	1	30	100	0	0	0	0	0	Isopropyl N-acetoxy-N-phenylcarbamate	C12H15NO4	Phenylcarbamic acid esters	237.1001	0	[M+H]+
HMDB0250759	1	30	100	0	0	0	0	0	L-Glutamic acid 5-benzyl ester	C12H15NO4	Amino acids, peptides, and analogues	237.1001	0	[M+H]+
HMDB0062175	1	30	100	0	0	0	0	0	N-Lactoylphenylalanine	C12H15NO4	Amino acids, peptides, and analogues	237.1001	0	[M+H]+
HMDB0013067	1	30	100	0	0	0	0	0	Salsoline-1-carboxylate	C12H15NO4	Tetrahydroisoquinolines	237.1001	0	[M+H]+

Figure S6. Application of the MS/MS validator in metabolite annotation. a) MS/MS validator tool. After identification by spectral matching the user can launch the MS/MS validator for additional metabolite MS/MS spectrum interpretation by clicking on the corresponding icon (left). As a result, the MS/MS validator allows the visualization of the fragment ions that match those observed experimentally (green peaks in the mirror spectra) (right). After approval of the identification result, the user can save and add the validated spectrum of the compound of interest to the database by clicking on the "floppy-disk" icon. b) Use of the MS/MS validator as a tool to reinspect ambiguous annotations, here applied to the feature 238.1074@10.41. From left to right the MS/MS spectrum of: 1-Carboxyethylphenylalanine, 3-Hydroxy-carbofuran, 4-Nitrophenyl hexanoate, 6,7-Dimethoxy-1,2,3,4-tetrahydroisoquinoline-3-carboxylic acid, Isopropyl N-acetoxy-N-phenylcarbamate, L-Glutamic acid 5-benzyl ester, N-Lactophenylalanine, and Sasoline-1-carboxylate. The green check highlights the most probable annotation results. Summary table of the identification results (bottom). The MS/MS spectrum can be not visualized in splitted view mode if the MS/MS validator doesn't identify any fragment ions.

2. Supplementary Tables

Table S1. Raw file data formats supported by MARS[(1)]

Vendor	Data format	Acquisition mode	IMS
Agilent	(.d)	DDA-based, DIA-based, Full Scan	yes
Waters	(.raw)	DDA-based, DIA-based, Full Scan	yes
Thermo	(.RAW)	DDA-based, DIA-based, Full Scan	no
Sciex	(.wiff)	DDA-based, DIA-based, Full Scan	no
Bruker[(2)]	(.d)	DDA-based, DIA-based, Full Scan	yes
Shimadzu	(.lcd)	DDA-based, DIA-based, Full Scan	no

[(1)] Agilent, Waters, and Shimadzu files can be directly imported while Thermo, Bruker, and Sciex files require the use of a converter downloadable from the instrument site, and the installation of additional vendor specific libraries stated in the software manual.[(2)] It includes FT-ICR.

Table S2. Levels associated to each identification adapted from Shymanski classification[2] with the description of minimum requirements (optional)

Levels	Description
1	Confirmed structure (by reference standard, match of MS, MS/MS, RT)
2a	Probable structure (by library spectrum match of MS and MS/MS, non-ambiguous assignment)
2b	Probable structure (assignment is ambiguous, so initially it was level 3, but the user can promote it to 2b by selecting a single assignment from the proposed solutions or by adding its own assignment).
3	Tentative candidate (there is an ambiguous match based on m/z in the DB (e.g. structural isomers) and the user cannot distinguish it using additional data (it cannot be converted to 2b)
4	Unequivocal molecular formula (use of the formula calculator)
5	Unknown compound (the m/z value at RT is annotated)

3. Downloadable Rule-based databases

DB for phytomics application (MARS-phytoDB)

Phytochemicals find applications in several fields, including the pharmaceutical[3] and food industries[4]. In nature, plants have one of the largest metabolome, with the number of phytochemicals is estimated to be greater than 30,000 for the average plant leaf.[5] Turi at al.[6] assumed the existence on earth of 300,000 to 350,000 plant species[7] and that each contains 4.7 unique metabolites, and calculated that only a small fraction of the estimated 1.4 to 1.5 million novel chemical structures belonging to the plant kingdom has been found. Therefore, rule-based approaches may prove useful to annotate species whose MS/MS spectra are still not stored in current repositories. A dataset of 23,713 phytochemicals was collected from four databases (KEGG, LipidMaps, HMDB, and PhenolExplorer) and classified into 10 main classes and 75 subclasses. Fragmentation rules were derived for 14 subclasses, and more than 10,000 metabolites belonging to these subclasses were fragmented in silico.

DB for nitrosamine detection (MARS-naDB)

Nitrosamine impurities are a great threat to human health. Indeed, this class of compounds can induce toxic effect even at extremely low concentration levels when delivered inside the organism, and some of them such as *N*-nitrosodimethylamine (NDMA), just one example, are recognised as powerful carcinogenic compounds.[8, 9] In addition, their simple formation from secondary amines through different synthetic routes can be seen in their occurrence in numerous different matrices. Indeed, nitrosamines can result from the activity of both bacteria and enzymes in the intestinal tract,[10-12] preservatives used in foods,[13-15] and manufacturing processes and chemical treatments of (raw) waters. Recently, the presence of NDMA and later of other *N*-nitroso compounds in some batches of pharmaceuticals was the cause of the withdrawal of drugs from the market (e.g., in sartans with tetrazole ring structure).[16, 17] The MARS-naDB in MARS is a virtual tandem MS-spectra library of nitrosamines that can be used to search for nitrosamines. A database of nitrosamine structures in .sdf format was first developed including a total of 27,944 compounds selected from different sources (i.e., 141 collected from Regulatory Agencies Safety Assessment, 129 nitrosamines commercially available from an E-molecules supplier, and 27,674 generated in silico by combining *N*-nitroso moiety with 235 specifically developed substituents). Following this, the structures included in linear and cyclic nitrosamines were also classified. Fragmentation rules were coded from literature[18-20] and in-house sources, and the whole database was fragmented accordingly.

4. Further details on MARS algorithms and tools

Template for the generation of a reference DB from in-house data

To generate a reference DB from in-house data using the MARS DB Manager, the user is asked to prepare a .csv file containing the following information: identification code (ID), common name, formula, exact mass, Level 1 classification, Level 2 classification, Level 3 classification, retention time, expected adducts, name of the instrument file with complete directory pathway (specifying positive or negative acquisition), and SMILES structures. Of these, the mandatory fields to be filled are identification code (ID), common name, formula, and exact mass. When instrument data files corresponding to in-house collections are no longer available, a MARS library can be generated from a .csv file without specifying the name of the raw files. However, MS/MS data are not supported in this case.

Pathway analysis

The 20 metabolic pathways available in MARS are the following: AAA biosynthesis; Alanine aspartate and glutamate metabolism; Arginine and proline metabolism; Arginine biosynthesis; Cysteine and methionine metabolism; Glycolysis and gluconeogenesis; GSH metabolism; Histidine metabolism; Lysine biosynthesis; Lysine degradation; N-glycan biosynthesis, Pentose phosphate pathway; Phenylalanine metabolism; Purine pathway; Pyrimidine metabolism; TCA cycle; Tryptophan metabolism; Tyrosine metabolism; Valine, leucine and isoleucine biosynthesis; Valine, leucine and isoleucine degradation. The MARS pathways were built by integrating data from literature and on-line sources (i.e., KEGG metabolic network[21-24] and PathBank pathways linked to HMDB)[25, 26]. For instance, in the Arginine and proline metabolism pathway five additional nodes were drawn compared to the KEGG pathway based on the study by Kwiatkowsky et al.[27] These authors studied purification in rat kidneys, identifying the biochemical purification of 4-oxo-L-proline reductase. This enzyme was partially characterized in 1962, and its molecular identity was solved recently. The above cited paper showed that 4-oxo-L-proline reductase metabolises 4-oxo-L-proline to cis-4-hydroxy-L-proline. Furthermore, additional information about the metabolism of glucoraphanin in the Cysteine and methionine metabolism pathway is provided. Glucoraphanin is a glucosinolate compound that derives from methionine and is associated with the health-promoting properties of broccoli. Although this molecule provides defence against pathogens and herbivore attack in plants, it is also associated with reduced risk for cardiovascular diseases and cancer in humans.[28, 29] Glucosinolate biosynthesis consists of up to three steps which are the following: (i) side chain elongation, (ii) core structure formation, and (iii) secondary modifications.[28] The synthesis of the glucoraphanin molecule begins with a chain stretching pathway of five enzymes that converts methionine into dihomomethionine and then into glucoraphanin through the action of seven enzymes.

The chain-elongation process for aliphatic glucosinolate synthesis begins with deamination of methionine to the corresponding 2-oxo acid. This reaction is catalysed by branched-chain amino acid aminotransferase (BCAT4). Although BCAT4 is localised in the cytosol, the rest of the enzymes involved in the elongation pathway are localised in the chloroplast.[30]

Identification Score Equations

The Overall Score (OS) is the weighted average of four partial scores:

$$OS = [x(S_M) + y(S_{IP}) + z(S_F) + w(S_{CCS})]/N$$

where:

-S_M: Mass score, proximity of the experimental mass with the theoretical mass of the proposed match;

-S_{IP}: Isotopic Pattern score, comparison of the experimental isotopic pattern abundance with the theoretical one of the proposed match (Isotopic Pattern Abundance score, S_{IPA}) and comparison of the experimental isotopic pattern spacing with the theoretical one of the proposed match (Isotopic Pattern Spacing score, S_{IPS}), combined as:

$$S_{IP} = \sqrt{S_{IPA} * S_{IPS}}$$

-S_F: Fragment score, evaluation of the number of matched fragment ions and their related intensity (I_{match}) compared to experimental fragment ions and their related intensity (I_{exp}) − i.e., matched fragments ions and top ten most intense unmatched fragments − detected in the acquired MS/MS spectrum for the proposed match. In particular, if their *m/z* difference is below the tuneable error tolerance, that fragment ion is included in the fragments score S_F calculated as:

$$S_F = 100 * \frac{\sum_1^n I_{match}}{\sum_1^m I_{exp}}$$

Notably, the pseudo-molecular fragment ion associated with the parent compound is not taken into consideration in the calculation of the S_F score as it does not provide information on the fragmentation of the molecule.

-S_{CCS}: Cross Collision Section score, proximity of the experimental CCS value to the one of the proposed match in the database (if CCS libraries are used and ion mobility data are analysed). By default, this score is set to 0.

-N: Sum of the weights (x+y+z+w)

MS/MS Validator

The MS/MS validator is not to be considered an in-silico fragmentation method but a tool for spectra rationalization. The encoded fragmentation tool takes the compound structure and generates fragments by breaking the bonds of the molecule 2 by 2. The number of generated fragments largely exceeds the ones chemistry can allow, but what drives here are the acquired experimental MS/MS spectra. This engine already proved useful in high-throughput applications for MetID and impurity analysis in pharmaceutical research (Mass-MetaSite and MassChemSite, two well-established software solutions used in numerous pharma companies).[31, 32] A validation of the approach was previously provided for Mass-MetaSite.[31] In **Figure S7,** the comparison of the MS/MS spectra identification of 5 metabolites using both experimental MS/MS library and the MS/MS validator is shown.

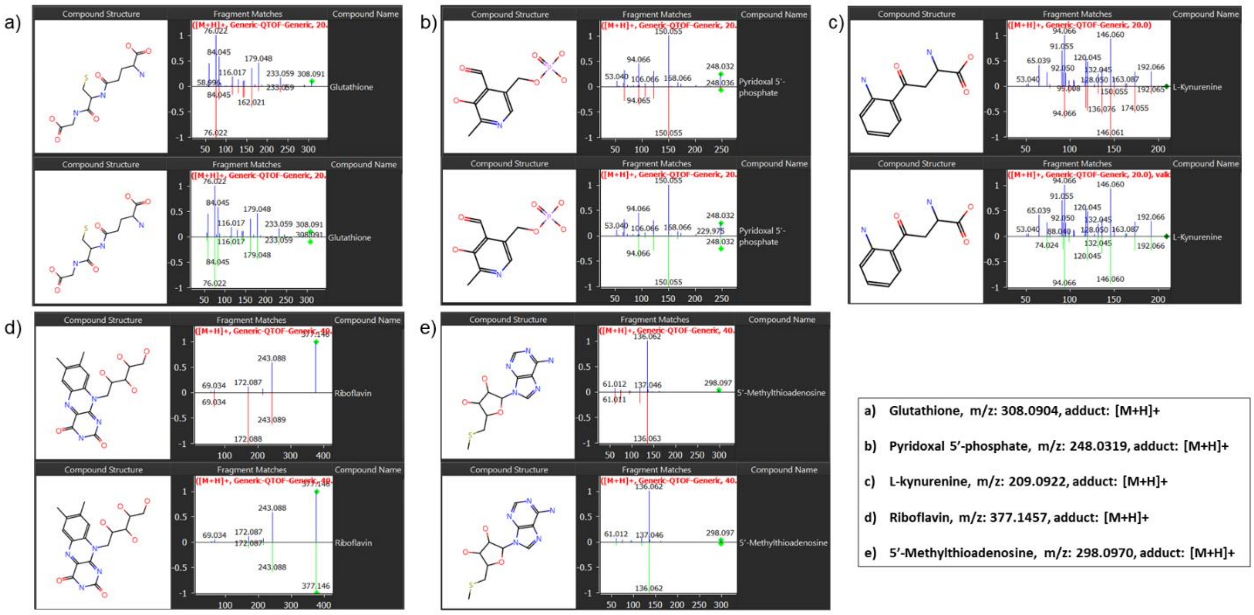

Figure S7. MS/MS spectra matching between experimental (blue), MS/MS HMDB library (red) and the MS/MS validator results (green) for glutathione, pyridoxal 5'-phosphate, L-kynurenine, riboflavin and, 5'-Methylthioadenosine.

5. Supplementary material CASE STUDY

Cell based assay

P1.HTR cells were transfected by electroporation with plasmid constructs coding for mouse indoleamine 2,3-dioxygenase 1 (IDO1; P1.IDO1). In their role as mock control cells, P1.HTR cells were transfected with the empty plasmid (P1.mock). Cells were cultured in Iscove's Modified

Dulbecco's Medium (Gibco, Invitrogen CA, USA) supplemented with 10% FCS (Gibco, Invitrogen CA, USA), 1 mM glutamine (Gibco, Invitrogen CA, USA), and penicillin/streptomycin (Gibco, Invitrogen CA, USA) at 37 °C in a humidified 7% CO_2 incubator. Stable transfectant cell lines were obtained by puromycin selection.

Extraction of metabolites

Cells were treated by using 200 µL of ACN/H_2O solution at 0°C containing 25 pg/µL of serotonin-d4, kynurenic acid-d5, and hydroxy-3-carboxyaldehyde-13C8, vortexed accurately and then placed on ice for 10 mins. After centrifugation at 14,000 rpm at 4°C for 30 min, an aliquot of 180 µL of the solution was transferred to a 1.5 mL Eppendorf micro test tube and dried at room temperature under nitrogen stream. The sample was solubilised with 45 µL of H_2O/CH_3OH solution (90/10, v/v) containing 100 pg/µL of tryptophan-d5 before LC-MS injection.

LC-MS method

Chromatography was performed on a Thermo Ultimate 3000 High Performance Liquid Chromatography system (San Jose, CA, USA). Analytes were separated on an Acquity-UPLC-BEH C18 column (150 × 2.1 mm; 1.7 µm – Waters) connected with a C18 guard column (2.1 × 5 mm; 1.7 µm – Waters). Mobile phases were water (A) and ACN (B) both containing 0.1% HCOOH. The gradient was initiated by using 0.5 % eluent B at 0.3 mL min^{-1} maintaining this condition for 2 min. In 12 min the eluent B increased to 35.0% and reached 99.0%, maintaining this condition for 2 min. The re-equilibration was 3 min for a total run time of 21 min. The column temperature was 40°C and the sample temperature was kept at 16°C. The injection volume was 1 µL. The mass analyser Q-Orbitrap (Q-Exactive, Thermo Scientific, San Jose, CA, USA) was equipped with a heated electrospray ionisation (HESI-II) source. The optimised HESI-II temperature was set at 320°C, the capillary temperature at 280°C, the electrospray voltage at 3.5 kV (positive mode), and S-lens value was adjusted to 50 V. Sheath and auxiliary gas were 45 and 15 arbitrary units, respectively. A Full MS/DDs (TopN) experiment was carried out to detect analytes. Settings for Full MS acquisition resolution, AGC target, Maximum It, and scan range were 35,000 (FWHM@200), 1e6, 150 ms, and 100-1000 m/z respectively. For DDs experiment, the resolution, AGC Target, Maximum IT, loop count, Isolation window, and stepped CE were set at 17,500 (FWHM@200), 5e5, 60 ms, 10, 1.0, and 12/25/50 respectively. For MS/MS trigger the value for minimum AGC target, Apex trigger, and dynamic exclusion were set at 4.00e3, 2 to 3 s, and 1 s. A preferred inclusion list, which also allowed other analytes to be picked, was used during the acquisition.

Data Analysis using MARS

Instrument raw data files were imported and converted in MARS (Molecular Discovery Ltd., UK, v. 1.0.0) by applying a MS signal filtering threshold of 5,000 and a MS/MS signal filtering threshold of 100. Then converted files were processed using a MS filtering threshold of 25,000 to let the gap-filler algorithm work in the range of 5,000-25,000. Peak smoothing was performed using the SDA algorithm set at "low". All of the other parameters were set at the default values, including the signal-to-noise filter, which was set at 3. Due to the reduced number of samples acquired in a single batch, the retention time correction was not activated. Metabolite annotation was performed using the HMDB database (version accessed on May 29th, 2023). Once imported in the MARS DB Manager module, 217,671 compounds were imported. The "All Metabolites" .xml file (version accessed on May 29th, 2023) was used to classify the compounds according to the HMDB classification and 50,365 MS/MS spectra were imported from the "MS-MS Spectra Files (XML) - Experimental" files (accessed on May 29th, 2023). It is noteworthy that during MS/MS import, the MARS DB Manager only imports spectra acquired using ITFT, QFT, Hybrid FT, QTOF, and ITTOF analyser MS-analysers, while the entire HMDB MS/MS file contains 65,030 spectra. Simply aiming to show one of the potential MARS workflows, no other options were activated. Metabolite annotation was performed by setting an MS tolerance of 5.0 ppm and a MS/MS tolerance of 20.0 ppm. The protonated molecule [M+H]+ was used in the first identification run while the investigation was extended in the second run to sodium [M+Na]+ adduct. In addition, the neutral losses of water or ammonia molecules were also explored.

6. Further information about MARS performances

Feature detection overlap among different software

To evaluate MARS feature detection performances, one pooled sample used as a quality control (QC) in the case study (available upon request) was analyzed also by using MZmine [v.3.9.0][33], MS-DIAL [v.5.1.230912][34], and XCMS-online [v.3.7.1][35]. For each software solution, the default parameters provided by each informatic tool to process Thermo data (e.g., Q-Exactive), were used to carry out the feature detection. Only the signal threshold value was manually set to 25,000 for each software. In addition, S/N=3 was set in MARS and XCMS-online while a similar setting was not found in MZmine and MS-DIAL, at least to our knowledge. By applying the following parameters during peak detection, the following Venn's diagram was generated (**Figure S8**).

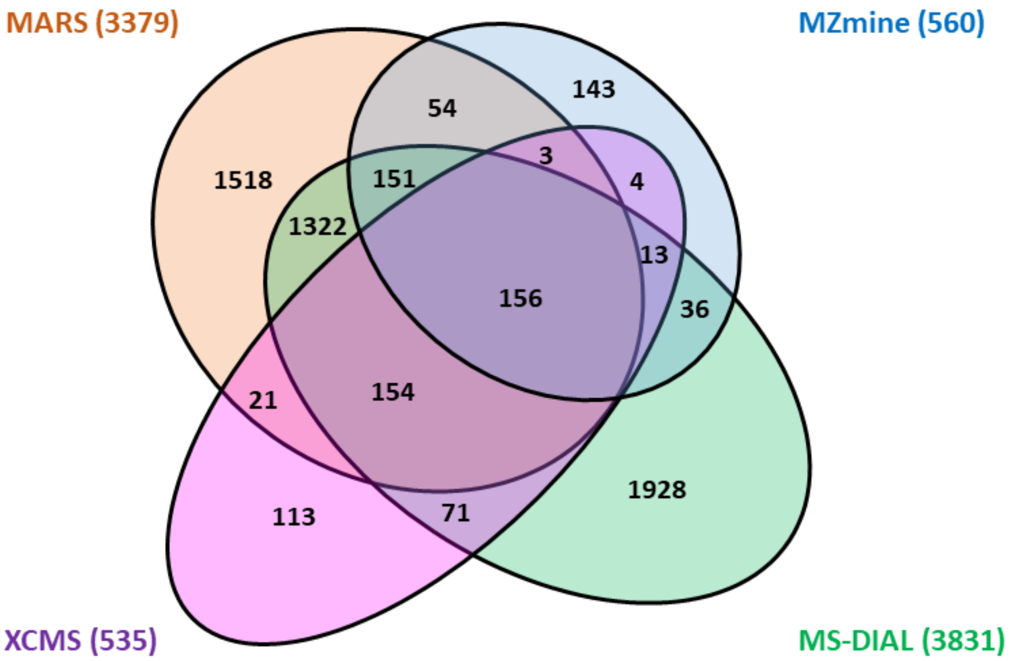

Figure S8: Overlapping of the chemical features (m/z@RT) detected by MARS, MZmine, MS-DIAL and XCMS-online for the one quality control (QC) sample analyzed in the case study. Sample alignment was performed using a m/z tolerance: 0.01 amu and a rt tolerance: 0.1 min.

It is noteworthy that while the authors are developing MARS, they have only basic knowledge with the other mentioned software solutions. Therefore, the setting and parameters used by us in XCMS, MZmine and MS-DIAL may not represent the identical condition of settings used with MARS in our case study.

Import of the MoNA (all) database in the MARS DB Manager

At the time of writing, the "all spectra" MoNA database (downloadable at https://mona.fiehnlab.ucdavis.edu/downloads) included 2,054,509 spectra, whose 210,156 spectra were from experimental data while 1,844,353 spectra were in silico generated.

MARS can import the MoNa (all spectra) library containing 2,054,509 spectra in 1h. Time refers to a laptop Dell Inspiron 14 5410 (x64bit) with processor 11th Gen Intel(R) Core(TM) i7-1195G7 @ 2.90GHz 1.80 GHz, RAM 16.0 GB. MARS imports the structure (SDF), MS, and MS/MS information collected in the MoNa library in a single step. During the import, MARS organizes the database not by spectra but by chemical structure. Thus, at the end of the import of the MoNa (all spectra) library, MARS imported 626,968 metabolites associated with 2,034,637 spectra; the remaining 19,872 spectra were discarded due to syntax error in the .sdf file.

It is noteworthy that MoNa (all spectra) collection contains experimental as well as in-silico predicted spectra for both LC-MS and GC-MS acquisitions. In addition, experimental spectra are collected from high- or low- resolution instruments, without distinction. Since MARS is a software developed for LC-MS based untargeted metabolomics with a preference for high-resolution instruments, MARS by default has a setting activated to import only high-resolution LC-MS and MS/MS data. In detail, spectra are imported or discarded based to the “instrument-type” and the “instrument” TAGs in the MoNA .sdf file. When this filter was selected, MARS imported 626,968 metabolites and 102,076 high-res MS/MS spectra in 50 min. Time refers to a laptop Dell Inspiron 14 5410 (x64bit) with processor 11th Gen Intel(R) Core(TM) i7-1195G7 @ 2.90GHz 1.80 GHz, RAM 16.0 GB.

Time required to detect features in one sample

The computational time required to perform the feature detection of one sample from the case study using the same LC-MS settings described in the methods section was 20 seconds. As an additional test, we performed the peak detection of 7 samples simultaneously. Indeed, when more than 2 samples are processed together to generate a data matrix additional algorithms like *RT correction and alignment, gap filler, and peak reintegration* are applied during the peak detection. In this case, the computational time required was 5 min. Time refers to a laptop Dell Inspiron 14 5410 (x64bit) with processor 11th Gen Intel(R) Core(TM) i7-1195G7 @ 2.90GHz 1.80 GHz, RAM 16.0 GB.

Search of MS/MS spectra against the MoNA database

The computational time required to perform the metabolite identification using the LC-MS Spectra (all) of MoNA High-res LC-MS spectra (17,552) on a sample containing 3,379 detected features is 3 min when excluding the computation level annotation 4 in the identification method. MARS provides the annotation of 811 metabolites at different confidence levels. The computational time required to perform the metabolite identification for the same sample including the level 4 computation is 7 min. Time refers to a laptop Dell Inspiron 14 5410 (x64bit) with processor 11th Gen Intel(R) Core(TM) i7-1195G7 @ 2.90GHz 1.80 GHz, RAM 16.0 GB.

Processing data on a large study

To demonstrate that MARS can work with larger datasets, the study ST002533 was downloaded from Metabolomics Workbench.[36] The dataset was composed of 178 samples which were analysed by LC-MS (reverse phase + acquisition in positive ion mode using a Thermo Q-Exactive HF-X Orbitrap). The raw data for the study ST002533 was downloaded, imported and processed using the default settings and a signal threshold of 20,000 both for conversion and for processing. The time for

conversion + data processing was 2.5 hours, with the detection of 74,640 features (**Figure S9**). Of them, 68,977 features were present in less of 80% of the samples (determined in 4 seconds by using the "Apply filter" option in MARS). When peak reintegration by column is applied, feature detection required additional 20 minutes. It is noteworthy that MARS has by default a spike filter activated, in which features described with 4 points (two points at the base and two additional points acquired) or less are automatically removed. This parameter is important to us to remove features whose chromatographic quality is low. Just to give an example, by reducing the spike filter value to 3, the number of detected features becomes 107,613. Data were run on laptop ASUS-Notebook N75VX (x64bit) with processor Intel(R) Core(TM) i7-6700HQ CPU @ 2.60GHz 2592 MHz, 4 cores, 8 logical processors, RAM 16.0 GB.

Samples | Data Matrix

Import MS/MS ... | Export Fingerprint ...

Uids	Sample	Compounds	MS/MS	Iso. Patt.	RT-min	RT-max	Labels	Type	Model Type	Comps not corr...	Area sum
168	SP_14	14060	3491	2514	0.0396605	16.0266		Sample	Training	0	2.69206e+11
169	SP_15	14221	3563	2645	0.0428544	16.0245		Sample	Training	0	2.70622e+11
170	SP_16	14422	3648	2682	0.0432956	15.9814		Sample	Training	0	2.8391e+11
171	SP_17	14730	3885	2778	0.0103169	16.025		Sample	Training	0	3.08604e+11
172	SP_18	14421	3744	2733	0.0287612	16.0517		Sample	Training	0	2.96279e+11
173	SP_19	14317	3817	2740	0	16.0185		Sample	Training	0	3.09095e+11
174	SP_20	14316	3853	2692	0.0429406	16.023		Sample	Training	0	3.09485e+11
175	SP_21	14701	3819	2839	0	16.0473		Sample	Training	0	3.05276e+11
176	SP_23	14718	3790	2775	0	16.0306		Sample	Training	0	3.10892e+11
177	SP_23_2	14787	3831	2778	0.034139	16.033		Sample	Training	0	3.10144e+11
178	SP_23_3	14704	3855	2815	0.0387763	16.0192		Sample	Training	0	3.08849e+11
	Supersample	74640	17093	17486	0	16.0375					3.79354e+11

	321.1205@12.07	888.0966@12.07	329.1378@12.07	544.3142@12.07	408.2518@12.07	176.0707@12.07	299.0577@12.07	183.0800@12.07
S_2	20.5315 / 0	0 / 0	0 / 0	0 / 0	20.1994 / 0	0 / 0	0 / 0	24.6997 / 35
S_3	20.6093 / 0	0 / 0	0 / 0	0 / 0	19.0884 / 0	0 / 0	0 / 0	24.6776 / 55
S_7	21.2969 / 0	0 / 0	0 / 0	0 / 0	0 / 0	0 / 0	18.5334 / 0	24.4515 / 47
S_11	19.8619 / 0	0 / 0	0 / 0	0 / 0	19.421 / 0	0 / 0	0 / 0	0 / 0
S_12	22.8917 / 20	0 / 0	0 / 0	0 / 0	0 / 0	0 / 0	18.6557 / 0	24.5488 / 59
S_14	20.4203 / 9	0 / 0	0 / 0	0 / 0	19.0739 / 0	0 / 0	0 / 0	24.2343 / 30
S_16	23.3461 / 16	0 / 0	0 / 0	0 / 0	0 / 0	0 / 0	0 / 0	24.6852 / 45
S_17	21.8155 / 10	0 / 0	0 / 0	0 / 0	19.461 / 0	0 / 0	0 / 0	24.69 / 56
S_19	22.9213 / 19	0 / 0	19.6806 / 0	0 / 0	19.5449 / 0	0 / 0	0 / 0	24.5719 / 58
S_20	20.9653 / 13	0 / 0	0 / 0	0 / 0	19.4891 / 0	0 / 0	0 / 0	24.1954 / 28
S_21	20.4164 / 0	0 / 0	0 / 0	0 / 0	20.3413 / 0	0 / 0	0 / 0	0 / 0
S_22	21.7424 / 0	0 / 0	0 / 0	0 / 0	19.8321 / 0	0 / 0	18.7452 / 0	24.5695 / 52
S_23	23.0806 / 27	0 / 0	0 / 0	0 / 0	19.5577 / 0	0 / 0	19.5062 / 0	24.883 / 61
S_25	21.4869 / 0	0 / 0	20.3453 / 0	0 / 0	0 / 0	0 / 0	19.9063 / 0	0 / 0
S_26	20.6388 / 0	0 / 0	19.7571 / 0	0 / 0	19.5232 / 0	0 / 0	0 / 0	24.5118 / 58
S_27	21.5463 / 13	0 / 0	0 / 0	0 / 0	19.3679 / 0	0 / 0	0 / 0	24.3706 / 54
S_30	19.9825 / 0	0 / 0	0 / 0	0 / 0	19.7661 / 0	0 / 0	18.5018 / 0	24.1291 / 46
S_31	20.2475 / 0	0 / 0	0 / 0	0 / 0	18.6982 / 0	0 / 0	18.973 / 0	24.3565 / 52
S_32	21.577 / 14	0 / 0	20.4796 / 0	0 / 0	19.0992 / 0	0 / 0	19.8338 / 0	24.5885 / 67
S_33	22.6719 / 14	0 / 0	0 / 0	0 / 0	0 / 0	0 / 0	20.3319 / 0	24.5557 / 62

Figure S9. Data from a large study (ST002533) processed in MARS.

References

1. Geller, S.; Lieberman, H.; Belanger, A. J.; Yew, N. S.; Kloss, A.; Ivanov, A. R., Comparison of Microflow and Analytical Flow Liquid Chromatography Coupled to Mass Spectrometry Global Metabolomics Methods Using a Urea Cycle Disorder Mouse Model. *J Proteome Res* **2022,** *21* (1), 151-163.
2. Schymanski, E. L.; Jeon, J.; Gulde, R.; Fenner, K.; Ruff, M.; Singer, H. P.; Hollender, J., Identifying small molecules via high resolution mass spectrometry: communicating confidence. *Environ Sci Technol* **2014,** *48* (4), 2097-8.
3. Bose, S.; Malik, J.; Mandal, S. C., Application of Phytochemicals in Pharmaceuticals. In *Advances in Pharmaceutical Biotechnology: Recent Progress and Future Applications*, Patra, J. K.; Shukla, A. C.; Das, G., Eds. Springer Singapore: Singapore, **2020,** 55-68.
4. Valverde, J., Industrial applications of phytochemicals. In *Handbook of Plant Food Phytochemicals*, **2013**, 473-501.
5. Kim, H. K.; Choi, Y. H.; Verpoorte, R., NMR-based plant metabolomics: where do we stand, where do we go? *Trends Biotechnol* **2011,** *29* (6), 267-75.
6. Turi, C. E.; Finley, J.; Shipley, P. R.; Murch, S. J.; Brown, P. N., Metabolomics for phytochemical discovery: development of statistical approaches using a cranberry model system. *J Nat Prod* **2015,** *78* (4), 953-66.
7. Miller, J. S., The Discovery of Medicines from Plants: A Current Biological Perspective1. *Economic Botany* **2011,** *65* (4), 396-407.
8. Barnes, J. M.; Magee, P. N., Some toxic properties of dimethylnitrosamine. *Br J Ind Med* **1954,** *11* (3), 167-74.
9. Magee, P. N.; Barnes, J. M., The production of malignant primary hepatic tumours in the rat by feeding dimethylnitrosamine. *Br J Cancer* **1956,** *10* (1), 114-22.
10. Stockbrugger, R. W.; Cotton, P. B.; Eugenides, N.; Bartholomew, B. A.; Hill, M. J.; Walters, C. L., Intragastric nitrites, nitrosamines, and bacterial overgrowth during cimetidine treatment. *Gut* **1982,** *23* (12), 1048-54.
11. Calmels, S.; Ohshima, H.; Bartsch, H., Nitrosamine formation by denitrifying and non-denitrifying bacteria: implication of nitrite reductase and nitrate reductase in nitrosation catalysis. *J Gen Microbiol* **1988,** *134* (1), 221-6.
12. Crews, C., The determination of N-nitrosamines in food. *Quality Assurance and Safety of Crops & Foods* **2010,** *2* (1), 2-12.
13. Tricker, A. R.; Preussmann, R., Carcinogenic N-nitrosamines in the diet: occurrence, formation, mechanisms and carcinogenic potential. *Mutat Res* **1991,** *259* (3-4), 277-89.
14. Herrmann, S. S.; Granby, K.; Duedahl-Olesen, L., Formation and mitigation of N-nitrosamines in nitrite preserved cooked sausages. *Food Chem* **2015,** *174*, 516-26.
15. Park, J. E.; Seo, J. E.; Lee, J. Y.; Kwon, H., Distribution of Seven N-Nitrosamines in Food. *Toxicol Res* **2015,** *31* (3), 279-88.
16. Karaman, I.; Ferreira, D. L.; Boulange, C. L.; Kaluarachchi, M. R.; Herrington, D.; Dona, A. C.; Castagne, R.; Moayyeri, A.; Lehne, B.; Loh, M.; de Vries, P. S.; Dehghan, A.; Franco, O. H.; Hofman, A.; Evangelou, E.; Tzoulaki, I.; Elliott, P.; Lindon, J. C.; Ebbels, T. M., Workflow for Integrated Processing of Multicohort Untargeted (1)H NMR Metabolomics Data in Large-Scale Metabolic Epidemiology. *J Proteome Res* **2016,** *15* (12), 4188-4194.
17. Karnovsky, A.; Li, S., Pathway Analysis for Targeted and Untargeted Metabolomics. *Methods Mol Biol* **2020,** *2104*, 387-400.
18. Lijinsky, W., *Chemistry and biology of N-nitroso compounds*. Cambridge University Press: 1992.
19. Zhao, Y. Y.; Boyd, J.; Hrudey, S. E.; Li, X. F., Characterization of new nitrosamines in drinking water using liquid chromatography tandem mass spectrometry. *Environ Sci Technol* **2006,** *40* (24), 7636-41.
20. Asare, S. O.; Hoskins, J. N.; Blessing, R. A.; Hertzler, R. L., Mass spectrometry based fragmentation patterns of nitrosamine compounds. *Rapid Commun Mass Spectrom* **2022,** *36* (8), e9261.
21. Kanehisa, M.; Goto, S., KEGG: kyoto encyclopedia of genes and genomes. *Nucleic Acids Res* **2000,** *28* (1), 27-30.

22. Kanehisa, M.; Goto, S.; Furumichi, M.; Tanabe, M.; Hirakawa, M., KEGG for representation and analysis of molecular networks involving diseases and drugs. *Nucleic Acids Res* **2010,** *38* (Database issue), D355-60.
23. Kanehisa, M.; Goto, S.; Hattori, M.; Aoki-Kinoshita, K. F.; Itoh, M.; Kawashima, S.; Katayama, T.; Araki, M.; Hirakawa, M., From genomics to chemical genomics: new developments in KEGG. *Nucleic Acids Res* **2006,** *34* (Database issue), D354-7.
24. Kanehisa, M.; Goto, S.; Sato, Y.; Kawashima, M.; Furumichi, M.; Tanabe, M., Data, information, knowledge and principle: back to metabolism in KEGG. *Nucleic Acids Res* **2014,** *42* (Database issue), D199-205.
25. Wishart, D. S.; Guo, A.; Oler, E.; Wang, F.; Anjum, A.; Peters, H.; Dizon, R.; Sayeeda, Z.; Tian, S.; Lee, B. L.; Berjanskii, M.; Mah, R.; Yamamoto, M.; Jovel, J.; Torres-Calzada, C.; Hiebert-Giesbrecht, M.; Lui, V. W.; Varshavi, D.; Varshavi, D.; Allen, D.; Arndt, D.; Khetarpal, N.; Sivakumaran, A.; Harford, K.; Sanford, S.; Yee, K.; Cao, X.; Budinski, Z.; Liigand, J.; Zhang, L.; Zheng, J.; Mandal, R.; Karu, N.; Dambrova, M.; Schioth, H. B.; Greiner, R.; Gautam, V., HMDB 5.0: the Human Metabolome Database for 2022. *Nucleic Acids Res* **2022,** *50* (D1), D622-D631.
26. Wishart, D. S.; Li, C.; Marcu, A.; Badran, H.; Pon, A.; Budinski, Z.; Patron, J.; Lipton, D.; Cao, X.; Oler, E.; Li, K.; Paccoud, M.; Hong, C.; Guo, A. C.; Chan, C.; Wei, W.; Ramirez-Gaona, M., PathBank: a comprehensive pathway database for model organisms. *Nucleic Acids Res* **2020,** *48* (D1), D470-D478.
27. Kwiatkowski, S.; Bozko, M.; Zarod, M.; Witecka, A.; Kocdemir, K.; Jagielski, A. K.; Drozak, J., Recharacterization of the mammalian cytosolic type 2 (R)-beta-hydroxybutyrate dehydrogenase as 4-oxo-l-proline reductase (EC 1.1.1.104). *J Biol Chem* **2022,** *298* (3), 101708.
28. Yang, H.; Liu, F.; Li, Y.; Yu, B., Reconstructing Biosynthetic Pathway of the Plant-Derived Cancer Chemopreventive-Precursor Glucoraphanin in Escherichia coli. *ACS Synth Biol* **2018,** *7* (1), 121-131.
29. Nugroho, A. B. D.; Lee, S. W.; Pervitasari, A. N.; Moon, H.; Choi, D.; Kim, J.; Kim, D. H., Transcriptomic and metabolic analyses revealed the modulatory effect of vernalization on glucosinolate metabolism in radish (Raphanus sativus L.). *Sci Rep* **2021,** *11* (1), 24023.
30. Kitainda, V.; Jez, J. M., Structural Studies of Aliphatic Glucosinolate Chain-Elongation Enzymes. *Antioxidants (Basel)* **2021,** *10* (9).
31. Zamora, I.; Fontaine, F.; Serra, B.; Plasencia, G., High-throughput, computer assisted, specific MetID. A revolution for drug discovery. *Drug Discov Today Technol* **2013,** *10* (1), e199-205.
32. Bonciarelli, S.; Desantis, J.; Goracci, L.; Siragusa, L.; Zamora, I.; Ortega-Carrasco, E., Automatic Identification of Lansoprazole Degradants under Stress Conditions by LC-HRMS with MassChemSite and WebChembase. *J Chem Inf Model* **2021,** *61* (6), 2706-2719.
33. Pluskal, T.; Korf, A.; Smirnov, A.; Schmid, R.; Fallon, T.; Du, X.; Weng, J.-K., CHAPTER 7. Metabolomics Data Analysis Using MZmine. **2020**, 232-254.
34. Tsugawa, H.; Ikeda, K.; Takahashi, M.; Satoh, A.; Mori, Y.; Uchino, H.; Okahashi, N.; Yamada, Y.; Tada, I.; Bonini, P.; Higashi, Y.; Okazaki, Y.; Zhou, Z.; Zhu, Z. J.; Koelmel, J.; Cajka, T.; Fiehn, O.; Saito, K.; Arita, M.; Arita, M., A lipidome atlas in MS-DIAL 4. *Nat Biotechnol* **2020,** *38* (10), 1159-1163.
35. Tautenhahn, R.; Patti, G. J.; Rinehart, D.; Siuzdak, G., XCMS Online: a web-based platform to process untargeted metabolomic data. *Anal Chem* **2012,** *84* (11), 5035-9.
36. Sud, M.; Fahy, E.; Cotter, D.; Azam, K.; Vadivelu, I.; Burant, C.; Edison, A.; Fiehn, O.; Higashi, R.; Nair, K. S.; Sumner, S.; Subramaniam, S., Metabolomics Workbench: An international repository for metabolomics data and metadata, metabolite standards, protocols, tutorials and training, and analysis tools. *Nucleic Acids Res* **2016,** *44* (D1), D463-70.
